# Association between cardiovascular risk factors and atrial fibrillation

**DOI:** 10.3389/fcvm.2023.1110424

**Published:** 2023-09-11

**Authors:** Guohao Wu, Jingguo Wu, Qin Lu, Yunjiu Cheng, Weiyi Mei

**Affiliations:** ^1^ Department of General Practice, Huadu District People's Hospital, Southern Medical University, Guangzhou, China; ^2^ Department of Emergency Medicine, The First Affiliated Hospital, Sun Yat-Sen University, Guangzhou, China; ^3^ Department of General Practice, The First Affiliated Hospital, Sun Yat-Sen University, Guangzhou, China; ^4^ Department of Guangdong Cardiovascular Institute, Guangdong Provincial People's Hospital (Guangdong Academy of Medical Sciences), Southern Medical University, Guangzhou, China; ^5^ Department of Cardiology, The First Affiliated Hospital, Sun Yat-Sen University, Guangzhou, China

**Keywords:** cardiovascular risk factors, atrial fibrillation, association, meta-analysis and systematic review, prospective cohort studies

## Abstract

**Background:**

The most prevalent sustained arrhythmia in medical practice, atrial fibrillation (AF) is closely associated with a high risk of cardiovascular disease. Nevertheless, the risk of AF associated with cardiovascular risk factors has not been well elucidated. We pooled all published studies to provide a better depiction of the relationship among cardiovascular risk factors with AF.

**Methods:**

Studies were searched in the MEDLINE, Web of Science, and EMBASE databases since initiation until January 15, 2022. Prospective cohort studies assessing the relationship a minimum of single cardiovascular risk factors to AF incidence were included if they contained adequate data for obtaining relative risks (RR) and 95% confidence intervals (CI). Random-effects models were utilized to perform independent meta-analyses on each cardiovascular risk factor. PROSPERO registry number: CRD42022310882.

**Results:**

A total of 17,098,955 individuals and 738,843 incident cases were reported for data from 101 studies included in the analysis. In all, the risk of AF was 1.39 (95% CI, 1.30–1.49) for obesity, 1.27 (95% CI, 1.22–1.32) per 5 kg/m^2^ for increase in body mass index, 1.19 (95% CI, 1.10–1.28) for former smokers, 1.23 (95% CI, 1.09–1.38) for current smokers, 1.31 (95% CI, 1.23–1.39) for diabetes mellitus, 1.68 (95% CI, 1.51–1.87) for hypertension, and 1.12 (95% CI, 0.95–1.32) for dyslipidemia.

**Interpretation:**

Adverse cardiovascular risk factors correlate with an increased risk of AF, yet dyslipidemia does not increase the risk of AF in the general population, potentially providing new insights for AF screening strategies among patients with these risk factors.

**Systematic Review Registration:**

https://www.crd.york.ac.uk/PROSPERO/, PROSPERO identifier (CRD42022310882).

## 1. Introduction

As the most prevalent arrhythmia diagnosed in medical practice (1), atrial fibrillation (AF) represents an enormous public health challenge with increasing clinical and public health expenses (2). The worldwide incidence in 2010 was evaluated at 5 million cases (3), whereas the prevalence in 2015 was evaluated at 33 million (4). There will be an expected rise in the prevalence of AF in people above 60 years of age from 3.9 million to 9 million by 2050 (5). Patients with AF are at high risk for stroke, thromboembolism and heart failure, leading to severe mortality and morbidity (6, 7). Costs to the economy caused by AF in the United States have been evaluated to exceed $6 billion per year (8). Therefore, primary prevention by changing risk factors is an important way to deal with the increased burden of atrial fibrillation (9).

Risk factors not subject to change, including age and gender, are non-controllable characteristics that have been shown to affect the occurrence of AF (10). As is well known, the general risk factors of cardiovascular disease comprise, obesity, smoking, dyslipidemia, diabetes and hypertension (11). Within prior studies, obesity, increment in body mass index (BMI), diabetes, and smoking have been proved to be related to elevated risk of AF (12–18). These meta-analyses, however, were only inclusive of studies released prior to 2017. Additional, data on dyslipidemia are finite and fairly disparate. Some observational studies unexpectedly found a “cholesterol paradox” in AF (19, 20), whereas others showed nonsignificant association (21–27). Contrary to expectations, certain observational studies revealed a counterintuitive link between blood cholesterol levels and atrial fibrillation risk, while a Mendelian randomization study involving individuals of European heritage failed to substantiate this correlation (28). Although the review showed that hypertension is a significant risk factor of AF (29), there is no available meta-analysis on the incidence of AF after hypertension based on prospective cohort studies.

Within this research, a systematic review and meta-analysis of prospective cohort studies were conducted performed to assess relationships among main cardiovascular risk factors, such as hypertension, diabetes mellitus, obesity, smoking, and dyslipidemia and the incidence of AF, focusing specifically on the intensity of effects of single risk factors.

## 2. Materials and methods

### 2.1. Search strategy

We conducted and reported this systematic review based on the prespecified standards (30) outlined by the PRISMA guidelines (31). The research program was registered with PROSPERO (number CRD42022310882). We systematically scoured the Web of Science, EMBASE, and MEDLINE databases from initiation until January 15, 2022, in search of studies regarding the relationship among cardiovascular risk factors with AF. The complete search strategies are provided on the appendix. All reference lists of previous meta-analyses, related reviews, and major reports were manually scoured to find additional matching studies.

### 2.2. Study selection

Abstracts and titles from searched articles were screened for eligibility independently by two reviewers (Wu G and Wu J). A third reviewer (Cheng Y) adjudicated disagreement. All studies deemed eligible based on title and abstract screening were reviewed for full text by two independent reviewers (Wu G, Wu J) using the same criteria. Discussion or involvement of a third reviewer (Cheng Y) was used to resolve inconsistent eligibility ratings. Studies were eligible for inclusion if they (1) were prospective cohort studies. (2) assessed the relationship for a minimum of one cardiovascular risk factor with occurrence of AF. (3) reported outcomes for hazard ratio (HR) or relative risk (RR) with 95% confidence intervals (CI), or offered adequate data to compute these outcomes. In case of a number of publications from an identical population, we included the most informative data (having enough baseline features and the most fully adjusted risk estimates). Exclusion criteria in detail can be found in the supplement.

### 2.3. Date extraction and quality assessment

Data were extracted individually by two authors (Wu G and Lu Q) using a standardized data extraction table. We extracted the following features from each qualifying study: name of the first author, year of publication, population source, geo-location, gender category, mean age at baseline, mean follow-up time, number of enrollees, number of incidents, approach of evaluating exposures and results, RR with respective 95% CIs, and adjusted covariates in multivariate analysis. The quality of included studies was assessed using the Newcastle-Ottawa scale (NOS) (32) for assessable items. In the present study, we regarded studies with score of six or above to be of high quality. Any disagreements were settled via consensus.

### 2.4. Data synthesis and analysis

The aggregated RRs were calculated using random effects models for each study's RR or HR. Random effects were utilized since these studies were performed in a broad setting with diverse population groups. This method would require that heterogeneity be taken into account while estimating aggregate effects. When the actual RRs were unavailable, the RRs and 95% CIs were calculated based on raw data. If RRs were usable, the most complete adjusted risk estimates presented in the publication were used. Pooled relative risks were expressed with 95% confidence intervals (CIs). We expressed continuous outcome data as weighted mean differences (WMDs) or mean differences (MDs) with 95% CIs. We used the Cochran's *χ*^2^ test to evaluate heterogeneity across studies, with quantification by the Cochran's *Q* and *I*^2^ statistics. A value in the range of 0–25% represented minimal heterogeneity, in the range of 26–75% indicated moderate heterogeneity, whereas in the range of over 75% denoted substantial heterogeneity (33).

The publication bias was tested using funnel plots and Egger's and Begg's regression tests (34, 35). Further publication bias was adjusted using Duval and Tweedie nonparametric trimming and filling procedures (36). To summarize, after assessing the number of studies in the asymmetric part, the approach removed the asymmetric edges of the funnel plot. To evaluate the actual center of the funnel by applying symmetric residual studies. Next, with the real center as the axis of symmetry, the studies pruned during the first step were first appended back to the trimmed funnel plot, while an equal number of predicted studies symmetrical to the trimmed studies were also appended back to the funnel plot. The ultimate composite estimation was derived in accordance with the filled funnel plot (37). We performed a number of sensitivity analyses to examine the robustness of the primary findings and to estimate possible sources of dissimilarity. To begin, fixed effects meta-analysis was conducted to evaluate the coherence of the primary outcomes of the random effects model. Next, to investigate the influence of study quality, sensitivity analyses were performed on significant quality ingredients, comprising covariates adjusted for in multivariate analyses (<3 factors or ≥3 factors), NOS scores (<6 or ≥6), average follow-up time (<10 or ≥10 years), case identification method (self-reported or measured), and subjective representativeness (occupation-based or population/community-based). Lastly, we excluded the two largest and outlier study estimates so as to investigate the impact of these studies on the aggregate RR. In all of our analyses, we utilized STATA, version 16.0 (Stata Corp LP, College Station, TX, USA). Bilateral *P* values <0.05 were deemed statistically significant.

## 3. Results

### 3.1. Study selection and baseline characteristics

**Figure 1:**
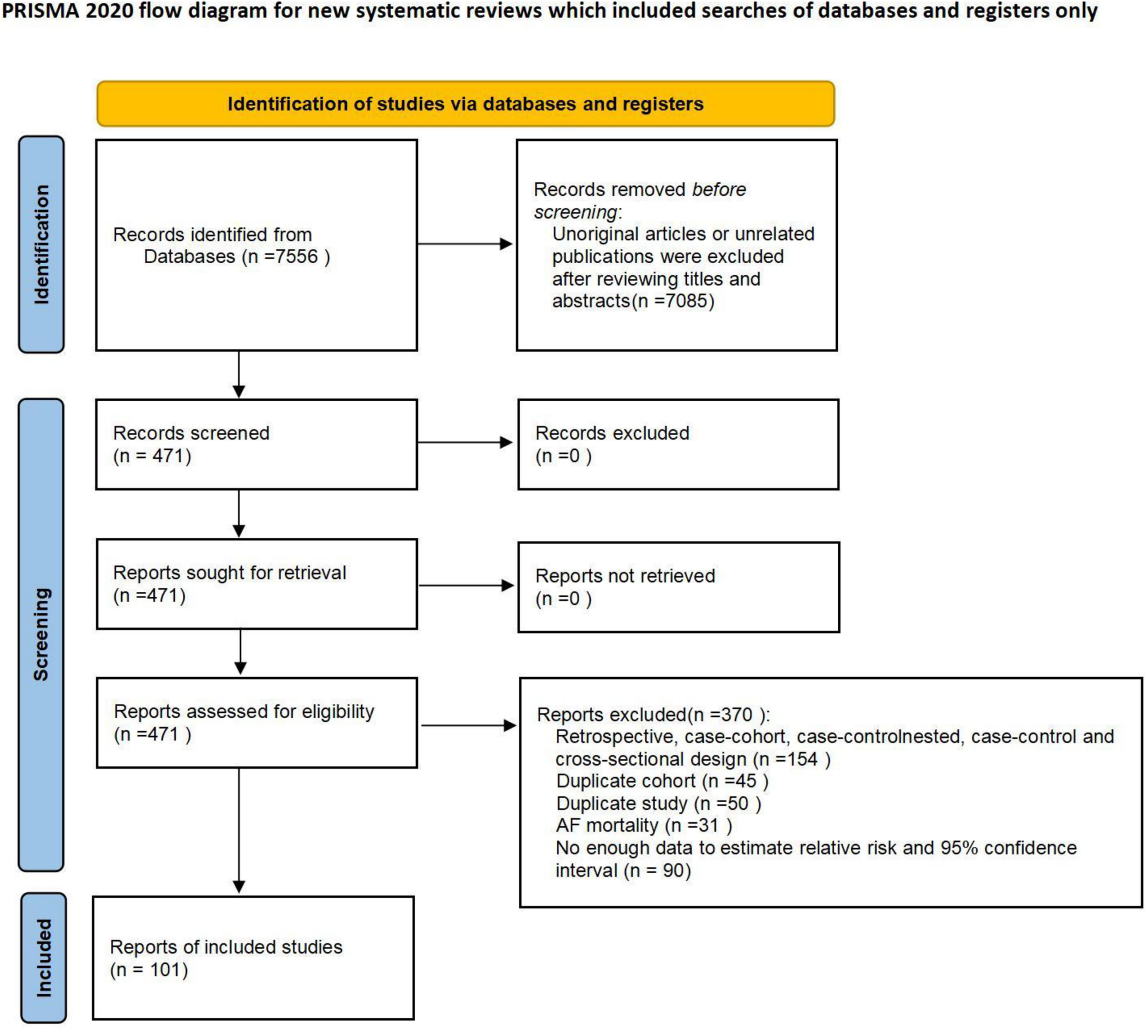
Flowchart of study selection.

**Figure 2:**
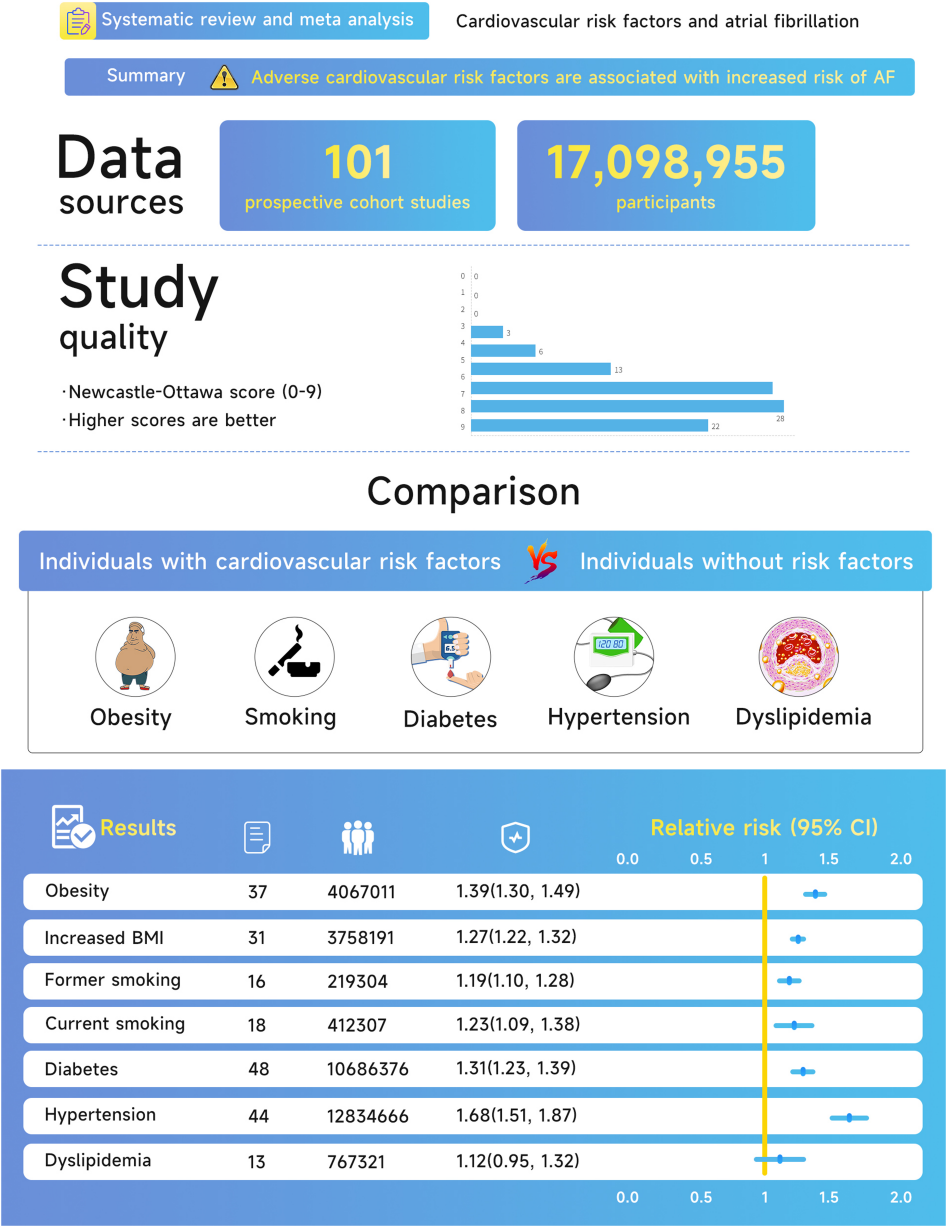
Central illustration of the association between cardiovascular risk factors and atrial fibrillation.

The systematic search of articles published before January 15, 2022, identified 7,556 results. After title and abstract screening, 471 articles were considered potentially relevant. Ultimately, 101 articles were included in the meta-analysis after a full-text review (Figure 1). The characteristics of the study are described in Table 1 while detailed information is presented in Supplementary Tables S1–S11. The typical results of this study are summarized in Figure 2. In total, 17, 098, 955 participants were included in this study to check the risk of AF in individuals with cardiovascular risk factors in comparison to those without, for a total of 738,843 AF cases. For all participants, the mean age was 56.6 years (each study ranged from 18.2 to 75.8 years). The studies selected were conducted and published among the years 1995 to 2021. 31 of these studies were carried out from North America, 49 from Europe, 17 from Asia, as well as 4 from Oceania.

**Table 1:** Characteristics of all studies included in the meta-analysis.

		Obesity	Increased BMI	Smoking	Diabetes	Hypertension	Dyslipidemia	Triglyceride	Total cholesterol	HDL-C	LDL-C
Number of studies		37	31	18	48	44	13	7	14	13	7
Published year		1995–2001	2001–2021	2005–2019	1995–2021	1989–2021	2009–2021	2008–2021	2008–2021	2008–2021	2013–2017
Location	Europe	18	17	7	21	19	5	3	5	4	3
	North America	11	8	4	16	13	4	0	5	4	2
	Asia	7	5	4	9	10	4	4	4	5	2
	Oceania	1	1	3	2	2	0	0	0	0	0
Sex	Men & women	28	23	16	42	37	12	7	11	12	6
	Men	5	5	1	4	5	0	0	2	0	0
	Women	4	3	1	2	2	1	0	1	1	1
Source	Population based	22	23	11	28	26	7	6	8	9	4
	Community-based	2	1	3	6	7	2	1	3	2	1
	Other	13	7	4	14	11	4	0	3	2	2
Mean follow-up (year)		12.1	13.1	10.6	10.1	10.6	6.3	11.0	13.1	11.3	10.8
Mean follow-up ≥10 year		19	21	9	22	20	0	3	8	7	3
Mean age (year)		52.7	56.4	60.3	57.4	57.4	56.3	60.1	58.8	60.8	57.7
Exposure confirmation	Measured	5	9	1	6	9	2	2	2	3	3
	Self-reported	1	0	1	1	1	1	0	0	0	0
	Measured/self-reported	28	22	16	33	27	9	5	12	10	4
	Other	3	0	0	8	7	1	0	0	0	0
Number of cases		71,793	70,451	19,907	543,572	634,988	20,484	3,422	7,626	7,852	3,162
Number of subjects		40,67,011	37,58,191	412,307	10,68,6376	12,83,4666	7,67,321	88,476	1,44,127	1,53,794	86,592
Newcastle-Ottawa score ≥6		34	29	17	35	39	11	7	14	13	7
Adjustment for 3 or more important confounding factors		27	26	15	35	31	9	3	10	7	4

BMI, body mass index; HDL-C, High-density lipoprotein cholesterol; LDL-C, Low-density lipoprotein cholesterol.

On the quality of research, 91% of enrolled studies were considered to be of excellent quality (NOS ≥ 6). There were 22 studies from occupational populations, 14 community-based researches, and 65 population-based researches among the 101 enrolled studies. Risk factor exposure was measured in 17 studies, self-reported in 2 studies, and established by additional approaches (combined physician diagnosis/medical history/present medication use/self-reported and measured data) in 82 studies. Adjusted RRs were provided for 87 studies, 77 of which adjusted for age and 76 for at least 3 significant comorbidity factors. Non-adjusted RRs were reported in 14 studies, and adjusted confounders were not available in a single study.

### 3.2. Obesity/BMI

**Figure 3:**
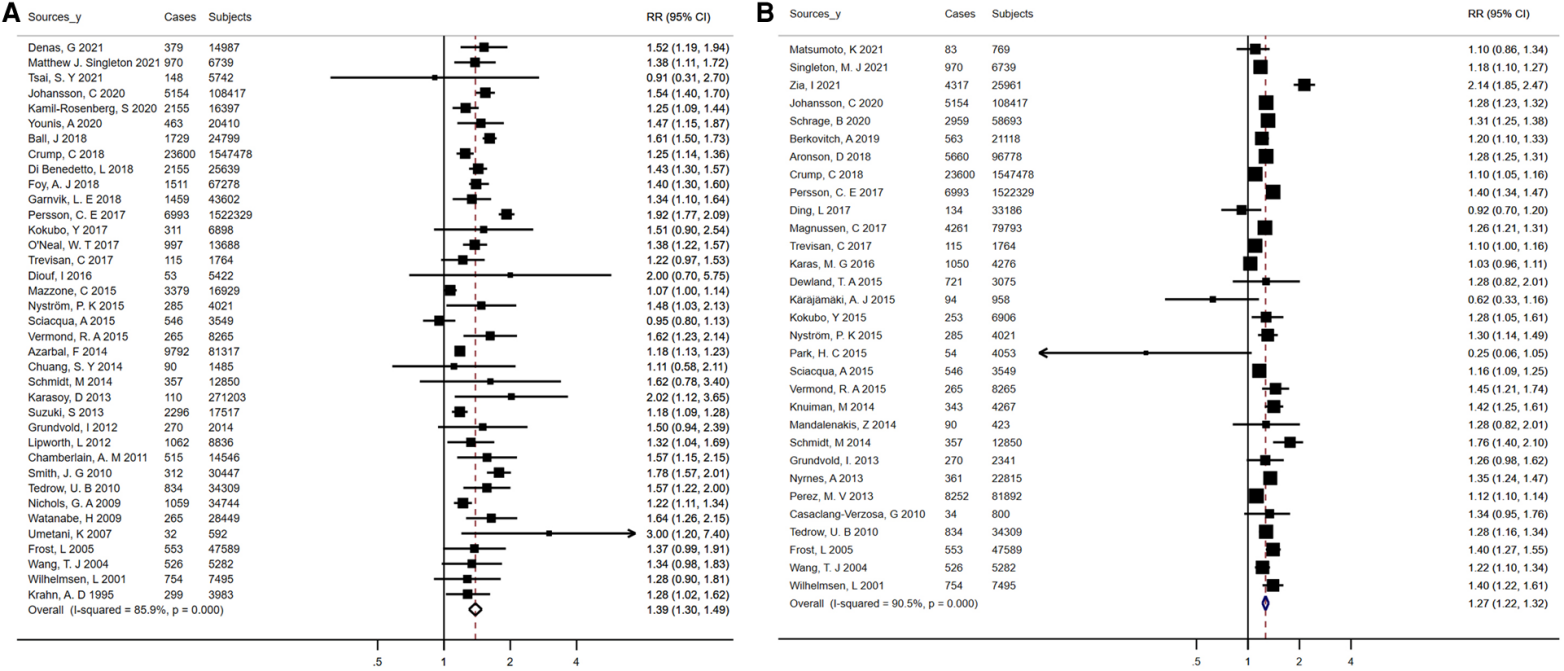
Forest plots for atrial fibrillation incidence (**A**) summary relative risks for obesity; (**B**) summary relative risks for Per 5 kg/m^2^ increase in body mass Index.

An aggregate of 5,824,726 participants with 120,266 events enrolled in the investigation of the relationship of obesity/BMI with AF risk. On the whole, patients with AF had a higher mean BMI than controls (MD, 0.74 kg/m^2^; 95% CI, 0.52 -0.95), and there was statistically significant heterogeneity between studies (*I*^2 ^= 90.2%; *P* < 0.01) (Supplementary figure S1). With a pooled RR on AF of 1.39 (95% CI, 1.30–1.49) for obesity and 1.27 (95% CI, 1.22–1.32) for each 5-unit increase in BMI, there was proof for a high degree of heterogeneity between the studies (*I*^2^ = 85.9% for obesity, *P* < 0.01; *I*^2^ = 90.5% for increased BMI, *P* < 0.01) (Figures 3A,B). After analyses of studies of excellent quality, studies enrolled in communities/populations, studies of average follow-up over 10 years, studies measuring height and weight, and fixed-effects models, there was no substantial variation in AF risk estimates associated with obesity or BMI, yet a high degree of heterogeneity remained (Supplementary Table S12). There was no measurable change in the pooled RR after excluding the two largest and outlier studies, and the estimated values for each case lay inside the confidence range of the aggregate estimate (Supplementary Table S12). Nevertheless, when the analysis of obesity and AF was limited to population/community-based studies, the summary RRs revealed no material variation, but significantly less heterogeneity (*I*^2 ^= 4.6%, *P* = 0.40). Neither funnel plot, Egger's test, nor Begg's test published evidence of bias (Supplementary Figures S3A,B).

### 3.3. Smoking

**Figure 4:**
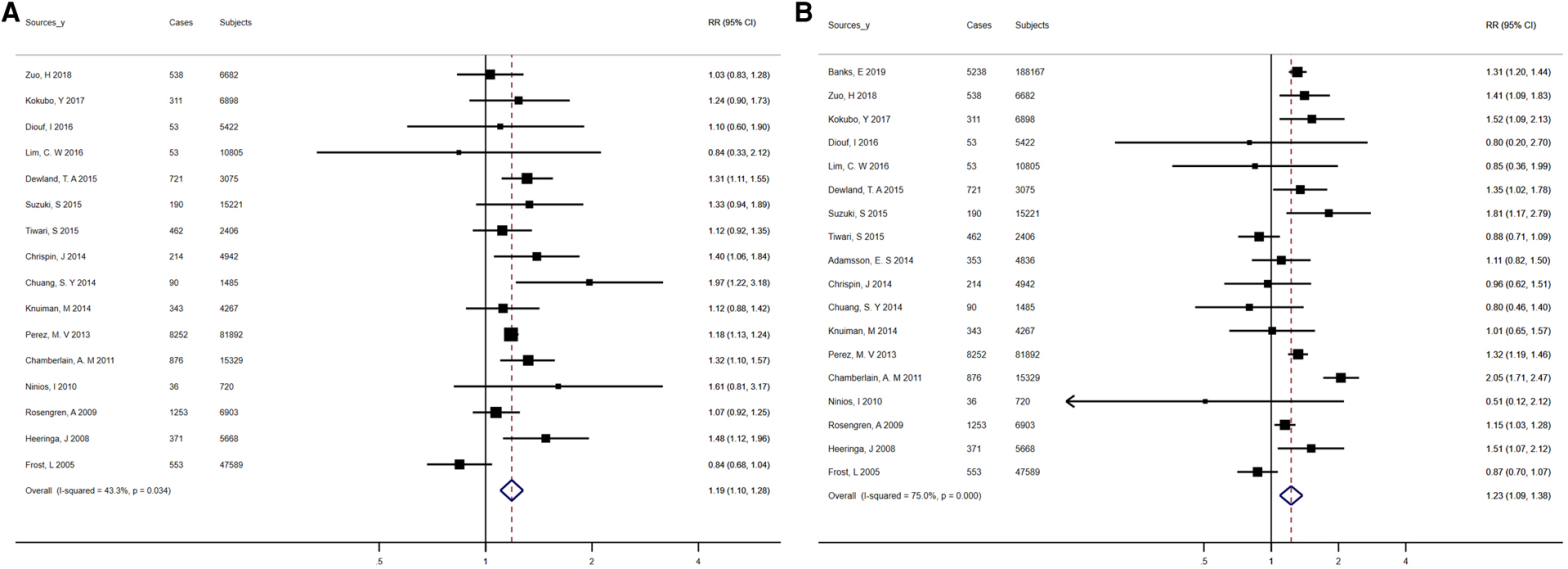
Forest plots for atrial fibrillation incidence (**A**) summary relative risks for former smokers vs. nonsmokers; (**B**) summary relative risks for current smokers versus nonsmokers.

This analysis included 18 studies reporting risk estimates for AF among former and current smokers as compared to never smokers, involving 412,307 participants and 19,907 events. Totally, for current smokers vs. never smokers and former smokers vs. never smokers, the pooled RRs were 1.23 (95% CI, 1.09–1.38) and 1.19 (95% CI, 1.10–1.28), separately (Figures 4A,B). Consistent increases in AF risk were observed among current and former smokers when analyses were repeated using fixed-effects models, comprising studies based on population/community, studies with an average follow-up exceeding 10 years, studies of high quality, and studies adjusted for over 3 confounders (Supplementary Table S14). Heterogeneity between studies was statistically significant in the analysis of current smokers, but partially not in the analysis of former smokers (*I*^2^ ranged from 59.5% to 89.8% for current smokers and 1.9% to 74.0% for former smokers, Supplementary Table S14). The sensitivity analysis showed no meaningful difference in RR after omitting an outlier study from the main analysis (Supplementary Table S14). Meanwhile, for current smokers (Egger's, *t* = − 0.79, *P* = 0.44; Begg's, *z* = 0.68, *P* = 0.50) and former smokers (Egger's, *t* = 0.49, *P* = 0.63; Begg's, *z* = 0.77, *P* = 0.44), no statistical proof of publication bias was available (data not shown).

### 3.4. Diabetes mellitus

**Figure 5:**
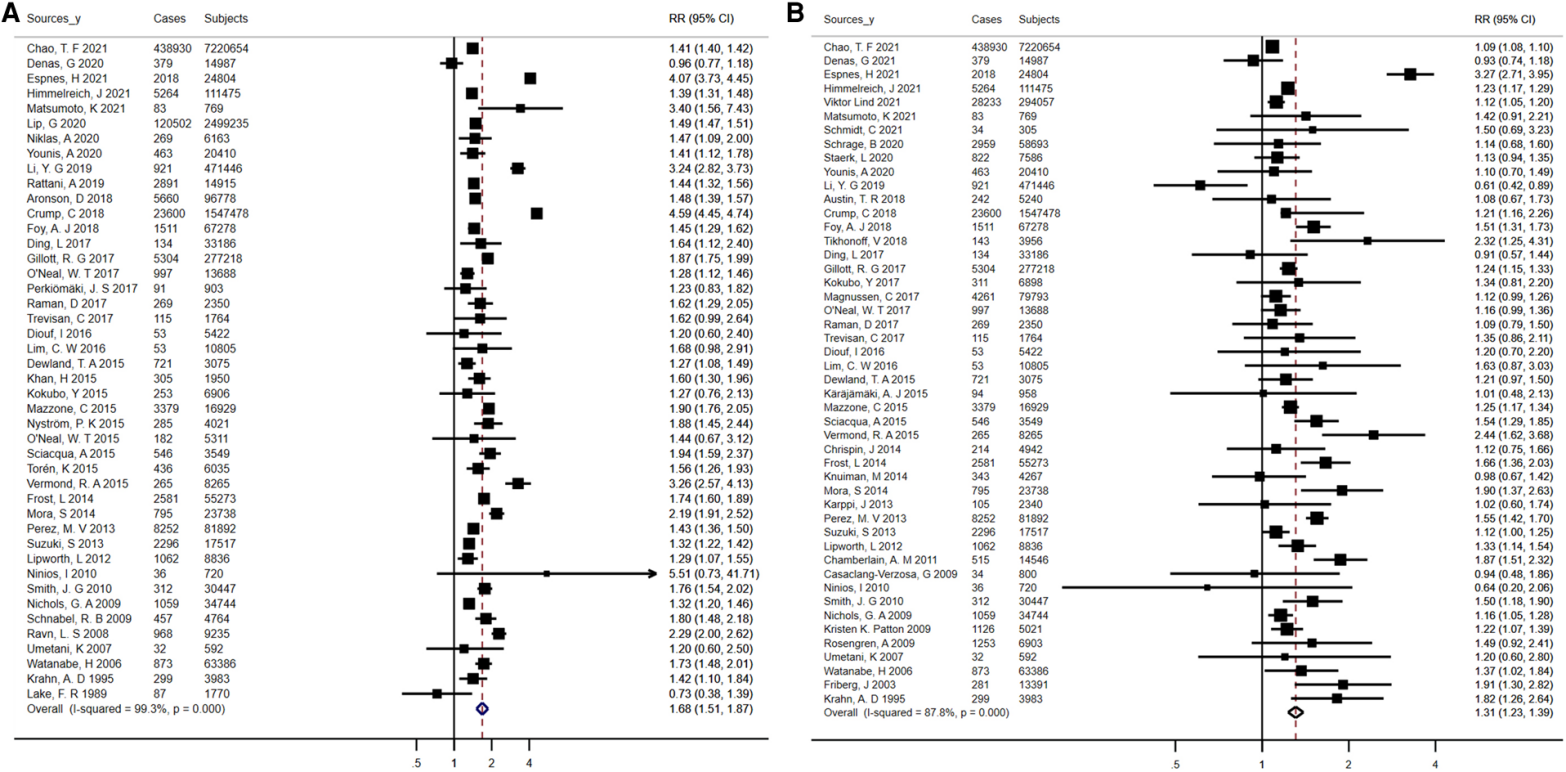
Forest plots for atrial fibrillation incidence (**A**) summary relative risks for diabetes; (**B**) summary relative risks for hypertension.

With regard to diabetes mellitus, there were forty-eight studies included in the analysis, which reported 543,572 events in 106,863,776 participants. The overall pooled RR for the risk of AF in association with diabetes was 1.31 (95% CI, 1.23–1.39), with a high degree of heterogeneity between studies (*I*^2^ = 87.8%, *P* < 0.01) (Figure 5A). The pooled RRs did not vary significantly following analysis using fixed-effects models, inclusion of studies with an average duration of follow-up over 10 years, population/community-based studies, or high-quality studies, with a high degree of heterogeneity among studies (Supplementary Table S15). Heterogeneity remained after exclusion of the two largest studies (*I*^2^ = 81.1%, *P* < 0.01), and sensitivity analysis omitting one outlier study from the main analysis showed no material variation in the results (Supplementary Table S15). Potential publication bias was noted based on asymmetric funnel plots, Egger's test (*t* = 4.12, *P* < 0.01) and Begg's test (*z* = 2.43, *P* = 0.02) (data not shown). For assessing the effect of underlying publication bias, the trim and fill approach along with 15 extra estimation studies were applied to balance the funnel plot and an adjusted summary random-effects RR was calculated, which showed a statistically significant relationship of diabetes and AF [RR = 1.12 (95% CI, 1.05–1.19)], indicating that when we considered the effect of publication bias, there was still a positive association (Supplementary Figure S4).

### 3.5. Hypertension

Reported 12,834,666 individuals and 634,988 events in 44 studies were included to assess the risk of AF among participants with hypertension. Altogether, compared with normotensive participants, hypertensive participants had an increased risk of AF [RR = 1.68 (95% CI, 1.51–1.87) for the random-effects model; RR = 1.51 (95% CI, 1.50–1.52) for the fixed-effects model] (Figure 5B). There was high heterogeneity among studies (*I*^2 ^= 99.3%, *P* < 0.01) (Supplementary Table S15). There was little variation in risk estimates based on the different exclusion and inclusion criteria, but heterogeneity remained, as indicated by the results of the sensitivity analysis (Supplementary Table S15). According to the Egger's test (*t* = 1.45, *P* = 0.15) or the Begg's test (*z* = 0.23, *P* = 0.82), no significant publication bias was observed (data not shown).

### 3.6. Dyslipidemia

**Figure 6:**
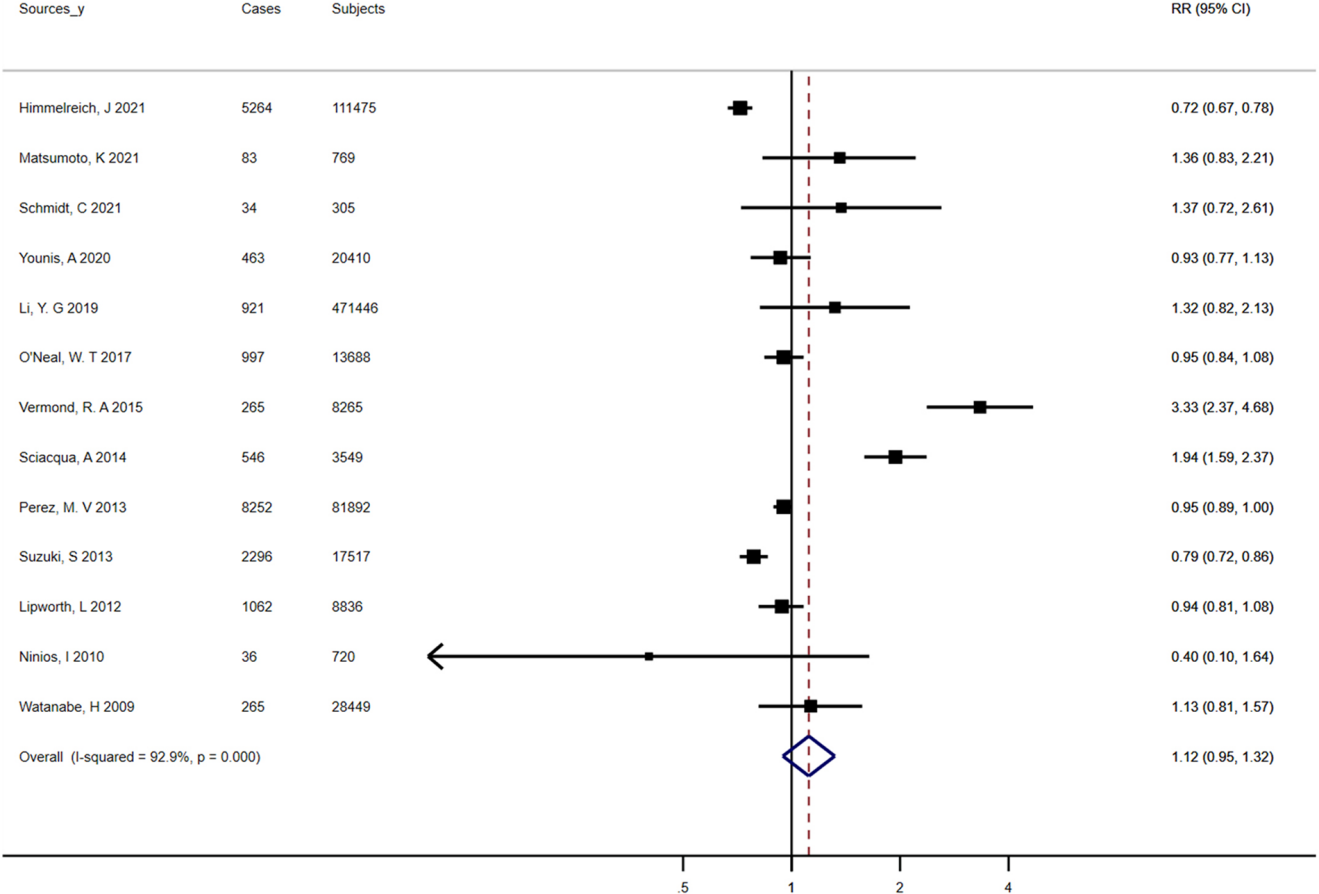
Forest plots for atrial fibrillation incidence (summary relative risks for dyslipidemia).

Involving 928,247 participants and 29,834 events, the analyses were performed to explore the relationship between AF prevalence and dyslipidemia. The overall pooled RR for the risk of AF associated with dyslipidemia was 1.12 (95% CI, 0.95–1.32), with a high degree of heterogeneity between studies (*I*^2^ = 92.9%, *P* < 0.01) (Figure 6). The mean levels of total cholesterol and triglycerides were not significantly related to AF (Supplementary Figures S2A,B). In addition, Low-density lipoprotein cholesterol (LDL-C) levels had no effect on the occurrence of AF (WMD, 0.02 mmol/L; 95% CI, −0.05 to 0.09), with statistically significant heterogeneity (*I*^2 ^= 61.8%; *P* = 0.02). High-density lipoprotein cholesterol (HDL-C) levels were on average 0.05 mmol/L (95% CI, −0.07 to −0.02) lower in patients with atrial fibrillation than in controls, again with significant heterogeneity (*I*^2 ^= 81.6%; *P* < 0.01). The results of sensitivity analyses showed no substantial change in the risk estimates for AF associated with dyslipidemia according to various exclusion and inclusion criteria, whereas heterogeneity persisted (Supplementary Table S16). When we repeated the analysis of total cholesterol and AF in studies with long-term follow-up (≥10 years), studies based on population/community, and studies of high quality, the pooled WMD remained statistically insignificant (Supplementary Table S17). Regarding the association between triglycerides and AF, the positive correlation was noted when we limited the meta-analysis to studies with a longer follow-up (≥10 years) (Supplementary Table S17). The negative association between HDL-C and AF persisted in analyses of studies based on population/community, high quality studies, and studies with an average follow-up over 10 years (*I*^2^ statistic values ranged from 72.7% to 87.7%), but disappeared in analyses of studies adjusted for at least 3 confounders and studies with large cohorts (Supplementary Table S18). No evidence of bias was published by funnel plot, Begg's test, or Egger's test (Supplementary Figures S5A,S6).

## 4. Discussion

The major risk factors for cardiovascular disease were discovered in association with enhanced risk of AF in this meta-analysis of a large sample size (over 17 million participants). The findings of our study broaden previous reports by showing not only that individual with well-established risk factors always underwent a heightened risk of AF, as well as demonstrating the relationship between AF and less well-defined risk factors. As far as we know, this is the first meta-analysis based entirely on prospective cohort studies summarizing literature on the association between hypertension with new-onset AF. Our pooled data reinforce findings from single studies. It is worth noting that there is no significant association between dyslipidemia and AF risk. Consistent to prior studies, the positive relationships among diabetes, smoking, increased BMI, obesity and AF were also demonstrated in our study. Despite the fact that obesity has been identified as a risk factor for AF, it is commonly found in conjunction with additional metabolic abnormalities, like dyslipidemia, hypertension and hyperglycemia, potentially mediating the relationship of obesity with the risk of AF (16, 38, 39). Recent studies found that both metabolically healthy and unhealthy obesity increase the risk of atrial fibrillation (40). Furthermore, there is a positive correlation between the cumulative burden of metabolic syndrome diagnostic criteria and its components over time and the risk of developing AF (41). In line to these discoveries, our study included obese individuals with a minimum of one metabolic abnormality and did observe a 39% increased risk of AF. In addition, the joint presence of risk factors probably related to additive impacts. Consequently, a thorough assessment of relevant risk factors is necessary when assessing the risk of AF.

Regarding hypertension, studies are numerous, but inconclusive. Several prospective cohort studies have shown that a strong risk factor for cardiovascular disease (CVD), including atrial fibrillation, is hypertension (29). Furthermore, in the study reported by Coccina F et al, both ambulatory and clinical systolic blood pressure prospectively predicted the onset of AF and daytime, nighttime, and 24-hour systolic blood pressure had similar correlations with future AF (42). Nevertheless, these studies covered a limited subgroup of population in general and were based on systolic blood pressure, potentially biasing the outcomes. Conversely, in a more general population, we indeed observed an increased risk of AF in those with pre-existing hypertension by 68%.

Despite our demonstration of an increased risk of AF in patients with hypertension and diabetes, it remains uncertain whether changing risk factors reduces the risk of AF. A meta-analysis conducted by Healey, J.S. et al. in 2005 included 11 randomized controlled human trials and demonstrated a 28% reduction in the risk of AF in angiotensin-converting enzyme inhibitor/angiotensin receptor blocker (ACEI/ARB) users compared to non-users (43). In addition, a 2015 meta-analysis showed that ACEI/ARBS could reduce the incidence of atrial fibrillation recurrence within 3 months and in long-term follow-up (44). For the treatment of diabetes, a meta-analysis based on 35 randomized controlled trials showed that sodium-glucose co-transport 2 (SGLT2) inhibitors significantly reduced the incidence of AF compared to placebo (45). Most recent meta-analyses also revealed that SGLT2 inhibitors were associated with a lower risk of AF (46, 47). These further suggest that hypertension and diabetes may be a cause of AF. However, recent evidence suggested a lack of significant protective effect of statins in the primary prevention of AF in the general population (48, 49), which also indicated that there was no apparent association between dyslipidemia and the risk of AF. With the exception of this, there was no causal relationship between total cholesterol, triglycerides, LDL cholesterol, HDL cholesterol and AF in the latest multivariate mendelian randomization study (50).

The specific bio-mechanisms behind these correlations are not completely clear, but can be attributed to some extent to the effects of structural (e.g., left atrial size), hemodynamic (e.g., left atrial stretch), electrical (e.g., altered conduction patterns due to atrial myocardial fibrosis), neurological (e.g., autonomic dysregulation), and inflammatory changes.

### 4.1. Inflammatory factors

It has been shown that accumulation of pericardial fat (51) and a systemic pro-inflammatory state produced by cytokines released from adipose tissue (52), as well as progressive atrial structural and electrical remodeling associated with obesity (53), may mediate the risk of atrial fibrillation.

### 4.2. Role of nicotine

Levels of nicotine in cigarettes produce an increase in plasma catecholamine levels (54), blood pressure and heart rate (55), and act as a potent inhibitor of cardiac A-type K^+^ channels. In addition, there is microRNA downregulation and transforming growth factor upregulation by nicotine, which leads to proarrhythmic atrial fibrosis (56).

### 4.3. Blood pressure and arterial stiffness

The relationship between increased blood pressure and atrial fibrillation exists for several explanations. It is directly related to the increase in left atrial diameter and blood pressure (57). Furthermore, measured by pulse pressure, increased arterial stiffness is associated with atrial fibrillation (58). Inflammation of the left atrium and arterial stiffness, which is associated with pulmonary venous fibrosis, which is the origin of atrial fibrillation, are potentially seen in people with elevated blood pressure, including pre-hypertension (59).

### 4.4. Insulin resistance and left ventricular hypertrophy

The cardiac autonomic neuropathy, similar to the peripheral autonomic neuropathy observed in diabetes, is a serious but neglected complication of diabetes (60, 61). Whereas, it is a hypothesis that cardiac autonomic dysfunction may be an important mechanism for the increased tendency of AF in diabetic patients. Added to this, insulin resistance is associated with an increased risk of left ventricular hypertrophy, which is itself a major risk factor for AF (62).

### 4.5. Dyslipidemia, cardiac load, and hyperthyroid status

Despite limited research on the mechanisms of dyslipidemia and AF, corresponding alterations in cardiac load (23), alterations in cardiac ion channels (63–65), and hyperthyroid status (66) may play a role. But whether this mechanism explains the observed association is hypothetical and requires further study.

Our findings may be useful in informing public health policy and allocating scarce resources for prevention. The RRs of each included study were pooled in our study to assess the intensity of the correlation, perform sensitivity analyses to examine the coherence of the relationship, and discuss potential biological mechanisms. In addition, the temporal nature of the prior exposure of the results was supported by the fact that all studies included in our analysis were prospective cohort designs. Moreover, the proof that treating several risk factors can decrease AF risk has been added. With this indicating that risk factors for CVD might as well be causative contributors to AF, more in-depth screening for AF in individuals having such risk factors is warranted.

To be acknowledged, the present meta-analysis was limited in the following aspects. First, we limited our analysis to individual risk elements, and there was a clear potential for the intensity of correlation to be weaker when multi-factor analysis was used. Second, there was substantial heterogeneity noted among the analyses of cardiovascular risk factors in spite of attempts at managing cross-study heterogeneity through proper meta-analysis techniques. However, in several sensitivity analyses, the risk estimates did not differ substantially, suggesting that heterogeneity may not affect the main results. Third, the potential for remnant or non-measured confounders could not be excluded, although the studies we included made attempts at controlling for a variety of identified risk factors. Fourth, some evidence suggested that publication bias was present solely within the analysis of diabetes. As shown in the funnel plot, there were some studies that lacked neutral or negative findings, indicating a potential overvaluation in the relationship. Fifth, the causal relationship cannot be determined in light of our present data based on observational studies, even though our results were robust and coherent across various sensitivity analyses. Sixth, cohorts of patients in some of the studies included in our meta-analysis were comparatively young and followed up for a short period of time, possibly contributing to the low prevalence of AF. The pooled RRs, nevertheless, did not materially vary when we performed repeated analyses of studies followed for over 10 years. Seventh, it was important to note that to extend such discoveries into African populations should be approached cautiously and would warrant additional inquiry, as our meta-analysis was primarily grounded in non-African research. Eighth, The studies we assessed might not have consistently differentiated between the various types of AF and diabetes. This disparity could lead to result discrepancies owing to variations in pathophysiological mechanisms and clinical implications within these subtypes. Future studies could enhance their insights by employing stratified analyses based on AF and diabetes subtypes.

Collectively, adverse cardiovascular risk factors were related to elevated risk of AF. Nevertheless, dyslipidemia does not increase the risk of AF. People with hypertension have a 68% increased relative risk of developing AF compared to those without hypertension, while obese patients had a 39% increased risk. In addition, current and former smoking were also associated with an increased risk of AF. Meanwhile, there is a need for caution in explaining the relationship of diabetes with AF, as publication bias is required to be considered.

## Data Availability

The original contributions presented in the study are included in the article/**Supplementary Material**, further inquiries can be directed to the corresponding authors.
