# Supplementary material for: Association between cardiovascular risk factors and atrial fibrillation
Source: Front Cardiovasc Med. 2023 Sep 11;10:1110424. doi: 10.3389/fcvm.2023.1110424 (PMC10518410; doi:10.3389/fcvm.2023.1110424)
Supplement: Supplementary file 1 [file Datasheet1.pdf]

Online-only supplements

Materials and methods

The systematic review and meta-analysis was conducted following the PRISMA guidelines. The protocol was published on the PROSPERO international prospective register of systematic reviews (registration number CRD42022310882).

Exclusion Criterion

We excluded studies that were not published as full reports, such as conference abstracts and letters to editors, studies that published as review, meta-analysis, studies of cross-sectional, case-cohort, case-control, nested case-control and retrospective design, studies that were done in populations with a history of cardiovascular disease or cancer, studies without sufficient data to estimate RR and 95% CIs, studies on AF mortality.

Definition of exposure

We defined a BMI of 30 kg/m^2 or more as “obesity” according to the WHO definition. Smoking status was classified into categories of “never”, “former” or “current”. Diabetes mellitus (DM) status included self-reported history of DM, current treatment of diabetes or physician diagnosed DM at enrollment. Hypertension was defined as being on anti-hypertensive drugs or systolic blood pressure ≥140/130 mm Hg or diastolic blood pressure ≥90/85 mm Hg. Dyslipidemia was defined as the levels of triglycerides, total cholesterol and low-density lipoprotein cholesterol (LDL-C) in the highest 25% and levels of high-density lipoprotein cholesterol (HDL-C) in the lowest 25%.

Date Extraction and Quality Assessment

For studies that reported results separately for men and women, but not combined, we combined the results using a fixed-effects model to generate an estimate for both sexes.

Search strategy

Our overall search strategy included the terms related to cardiovascular risk factors (“Body mass index”, “Obesity”, “Hypertension”, “Smoking”, “Diabetes Mellitus”, “Triglycerides”, “Cholesterol”, “Dyslipidemia”), combined with terms of AF (“atrial fibrillation”, “auricular fibrillation”, “fibrillations auricular”) as follows.

("epidemiologic studies"[MeSH Terms] OR "cohort studies"[MeSH Terms] OR "epidemiologic"[Text Word] OR "cohort"[Text Word] OR "longitudinal"[Text Word] OR "follow up"[Text Word] OR "observational"[Text Word] OR "prospective"[Text Word] OR ("epidemiologic studies"[Title/Abstract] OR "cohort studies"[Title/Abstract])) AND ("atrial fibrillation"[MeSH Terms] OR "atrial fibrillation"[Title/Abstract] OR "atrial fibrillations"[Title/Abstract] OR "fibrillation atrial"[Title/Abstract] OR "fibrillations atrial"[Title/Abstract] OR "auricular fibrillation"[Title/Abstract] OR "auricular fibrillations"[Title/Abstract] OR (("fibril"[All Fields] OR "fibril s"[All Fields] OR "fibrilation"[All Fields] OR "fibrilization"[All Fields] OR "fibrilized"[All Fields] OR "fibrillate"[All Fields] OR "fibrillated"[All Fields] OR "fibrillates"[All Fields] OR "fibrillating"[All Fields] OR

"Fibrillation"[All Fields] OR "Fibrillations"[All Fields] OR "fibrillization"[All Fields] OR "fibrillize"[All Fields] OR "fibrillized"[All Fields] OR "fibrillizes"[All Fields] OR "fibrillizing"[All Fields] OR "fibrillous"[All Fields] OR "fibrills"[All Fields] OR "fibrils"[All Fields]) AND "Auricular"[Title/Abstract]) OR "fibrillations auricular"[Title/Abstract] OR "persistent atrial fibrillation"[Title/Abstract] OR "atrial fibrillation persistent"[Title/Abstract] OR (("atrial fibrillation"[MeSH Terms] OR ("Atrial"[All Fields] AND "Fibrillation"[All Fields]) OR "atrial fibrillation"[All Fields] OR ("Atrial"[All Fields] AND "Fibrillations"[All Fields]) OR "atrial fibrillations"[All Fields]) AND "Persistent"[Title/Abstract]) OR "fibrillation persistent atrial"[Title/Abstract] OR (("fibril"[All Fields] OR "fibril s"[All Fields] OR "fibrilation"[All Fields] OR "fibrilization"[All Fields] OR "fibrilized"[All Fields] OR "fibrillate"[All Fields] OR "fibrillated"[All Fields] OR "fibrillates"[All Fields] OR "fibrillating"[All Fields] OR "Fibrillation"[All Fields] OR "Fibrillations"[All Fields] OR "fibrillization"[All Fields] OR "fibrillize"[All Fields] OR "fibrillized"[All Fields] OR "fibrillizes"[All Fields] OR "fibrillizing"[All Fields] OR "fibrillous"[All Fields] OR "fibrills"[All Fields] OR "fibrils"[All Fields]) AND "persistent atrial"[Title/Abstract]) OR "persistent atrial fibrillations"[Title/Abstract] OR "familial atrial fibrillation"[Title/Abstract] OR "atrial fibrillation familial"[Title/Abstract] OR (("atrial fibrillation"[MeSH Terms] OR ("Atrial"[All Fields] AND "Fibrillation"[All Fields]) OR "atrial fibrillation"[All Fields] OR ("Atrial"[All Fields] AND "Fibrillations"[All Fields]) OR "atrial fibrillations"[All Fields]) AND "Familial"[Title/Abstract]) OR (("familialities"[All Fields] OR "familiality"[All Fields] OR "familially"[All Fields] OR "familials"[All Fields] OR "familie"[All Fields] OR "family"[MeSH Terms] OR "family"[All Fields] OR "Familial"[All Fields] OR "families"[All Fields] OR "family s"[All Fields] OR "familys"[All Fields]) AND "atrial fibrillations"[Title/Abstract]) OR (("fibril"[All Fields] OR "fibril s"[All Fields] OR "fibrilation"[All Fields] OR "fibrilization"[All Fields] OR "fibrilized"[All Fields] OR "fibrillate"[All Fields] OR "fibrillated"[All Fields] OR "fibrillates"[All Fields] OR "fibrillating"[All Fields] OR "Fibrillation"[All Fields] OR "Fibrillations"[All Fields] OR "fibrillization"[All Fields] OR "fibrillize"[All Fields] OR "fibrillized"[All Fields] OR "fibrillizes"[All Fields] OR "fibrillizing"[All Fields] OR "fibrillous"[All Fields] OR "fibrills"[All Fields] OR "fibrils"[All Fields]) AND "familial atrial"[Title/Abstract]) OR (("fibril"[All Fields] OR "fibril s"[All Fields] OR "fibrilation"[All Fields] OR "fibrilization"[All Fields] OR "fibrilized"[All Fields] OR "fibrillate"[All Fields] OR "fibrillated"[All Fields] OR "fibrillates"[All Fields] OR "fibrillating"[All Fields] OR "Fibrillation"[All Fields] OR "Fibrillations"[All Fields] OR "fibrillization"[All Fields] OR "fibrillize"[All Fields] OR "fibrillized"[All Fields] OR "fibrillizes"[All Fields] OR "fibrillizing"[All Fields] OR "fibrillous"[All Fields] OR "fibrills"[All Fields] OR "fibrils"[All Fields]) AND "familial atrial"[Title/Abstract]) OR "paroxysmal atrial fibrillation"[Title/Abstract] OR "atrial fibrillation paroxysmal"[Title/Abstract] OR "atrial fibrillations paroxysmal"[Title/Abstract] OR "fibrillation paroxysmal atrial"[Title/Abstract] OR "fibrillations paroxysmal atrial"[Title/Abstract] OR "paroxysmal atrial fibrillations"[Title/Abstract]) AND ("Body Mass Index"[MeSH Terms] OR "BMI"[Title/Abstract] OR "index body mass"[Title/Abstract] OR "quetelet index"[Title/Abstract] OR "index quetelet"[Title/Abstract] OR "quetelet s index"[Title/Abstract] OR "quetelets index"[Title/Abstract] OR ("Obesity"[MeSH Terms] OR "obese"[Title/Abstract] OR "adiposity"[Title/Abstract] OR "overweight"[Title/Abstract] OR "bodyweight"[Title/Abstract] OR "Body Mass Index"[Title/Abstract] OR "body fat"[Title/Abstract] OR "body fat mass"[Title/Abstract]) OR ("Hypertension"[MeSH Terms] OR "blood pressure high"[Title/Abstract] OR "blood pressures high"[Title/Abstract] OR "high blood pressure"[Title/Abstract] OR "high blood pressures"[Title/Abstract] OR "arterial

hypertension"[Title/Abstract]) OR ("Smoking"[MeSH Terms] OR "smoking behaviors"[Title/Abstract] OR "behavior smoking"[Title/Abstract] OR "behaviors smoking"[Title/Abstract] OR "smoking behavior"[Title/Abstract] OR "smoking habit"[Title/Abstract] OR "habit smoking"[Title/Abstract] OR "habits smoking"[Title/Abstract] OR "smoking habits"[Title/Abstract]) OR ("Triglycerides"[MeSH Terms] OR "Triacylglycerol"[Title/Abstract] OR "Triacylglycerols"[Title/Abstract]) OR ("lipoproteins, hdl"[MeSH Terms] OR "hdl lipoproteins"[Title/Abstract] OR "heavy lipoproteins"[Title/Abstract] OR (("lipoprotein s"[All Fields] OR "lipoproteine"[All Fields] OR "Lipoproteins"[MeSH Terms] OR "Lipoproteins"[All Fields] OR "Lipoprotein"[All Fields]) AND "Heavy"[Title/Abstract]) OR "high density lipoproteins"[Title/Abstract] OR "high density lipoproteins"[Title/Abstract] OR "lipoproteins high density"[Title/Abstract] OR "alpha-Lipoproteins"[Title/Abstract] OR "alpha-Lipoproteins"[Title/Abstract] OR "alpha 1 lipoprotein"[Title/Abstract]) OR ("cholesterol, ldl"[MeSH Terms] OR "low density lipoprotein cholesterol"[Title/Abstract] OR "beta lipoprotein cholesterol"[Title/Abstract] OR "cholesterol beta lipoprotein"[Title/Abstract] OR "beta lipoprotein cholesterol"[Title/Abstract] OR "ldl cholesterol"[Title/Abstract] OR "cholesteryl linoleate ldl"[Title/Abstract] OR "ldl cholesteryl linoleate"[Title/Abstract]) OR ("Diabetes Mellitus"[MeSH Terms] OR "Diabetes"[Title/Abstract]) OR "total cholesterol"[Title/Abstract] OR ("Obesity"[Title/Abstract] OR "Body Mass Index"[Title/Abstract] OR "Hypertension"[Title/Abstract] OR "Smoking"[Title/Abstract] OR "Triglycerides"[Title/Abstract] OR "lipoproteins hdl"[Title/Abstract] OR "cholesterol ldl"[Title/Abstract] OR "Diabetes Mellitus"[Title/Abstract] OR "total cholesterol"[Title/Abstract]))

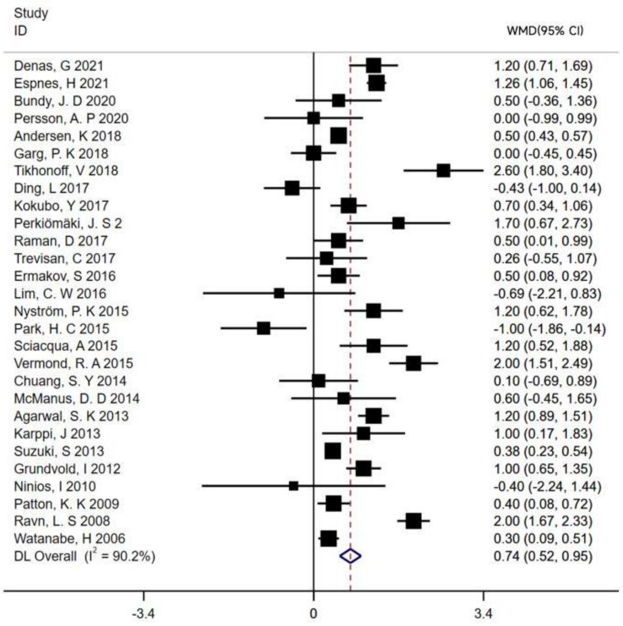

efigure 1 The influence of BMI levels (kg/m^2) on AF.

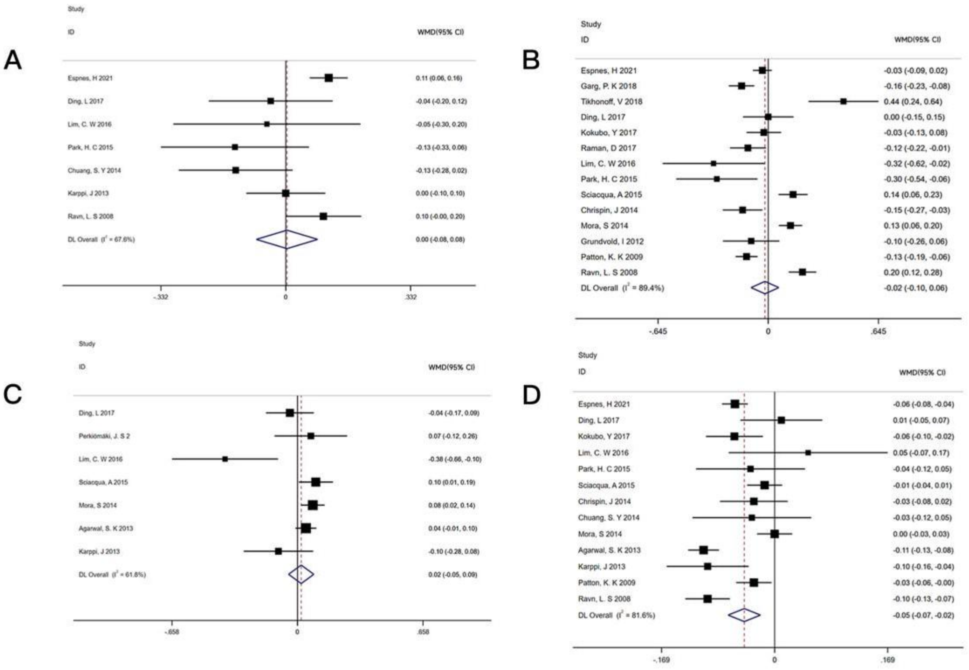

efigure 2 (A) The influence of triglyceride levels (mmol/L) on VTE; (B) The influence of total cholesterol levels (mmol/L) on AF; (C) The influence of LDL cholesterol levels (mmol/L) on AF.; (D) The influence of HDL cholesterol levels (mmol/L) on AF; AF: atrial fibrillation.

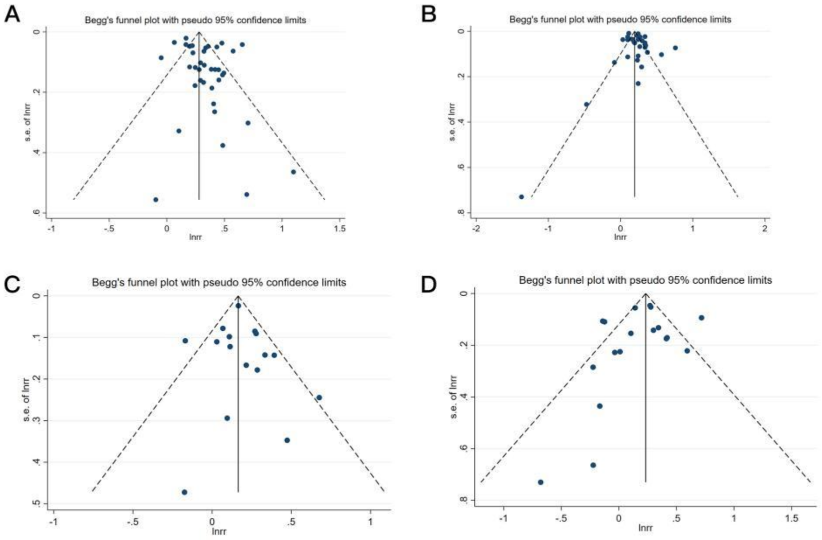

efigure 3 (A) Funnel Plots showing association between obesity and AF; (B) Funnel Plots showing association between increased BMI and AF; (C) Funnel Plots showing association between former smoking and AF; (D) Funnel Plots showing association between current smoking and AF; BMI: Body mass index; AF: atrial fibrillation.

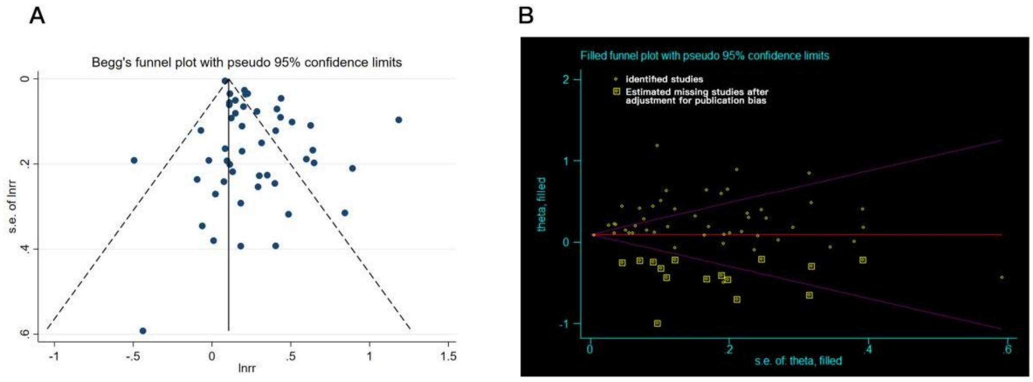

efigure 4 Funnel Plots showing Association between Diabetes and AF without and with Trim and Fill.

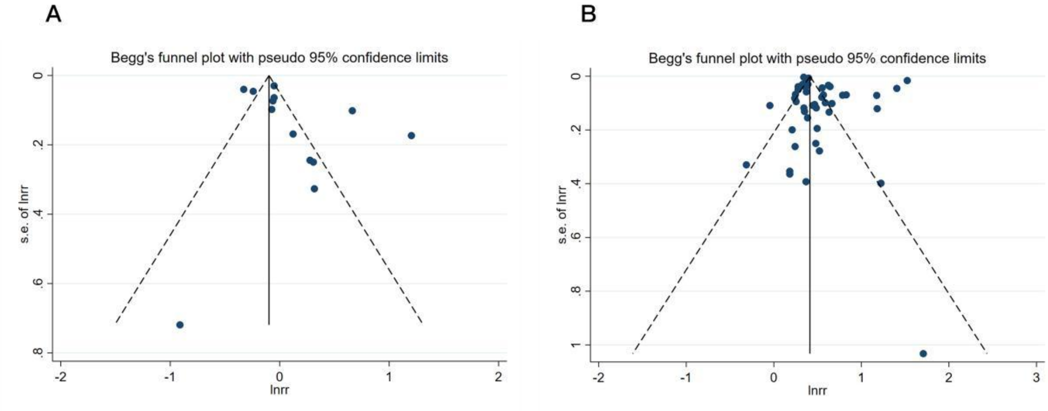

efigure 5 (A) Funnel Plots showing association between dyslipidemia and AF; (B) Funnel Plots showing association between hypertension and AF; AF: atrial fibrillation.

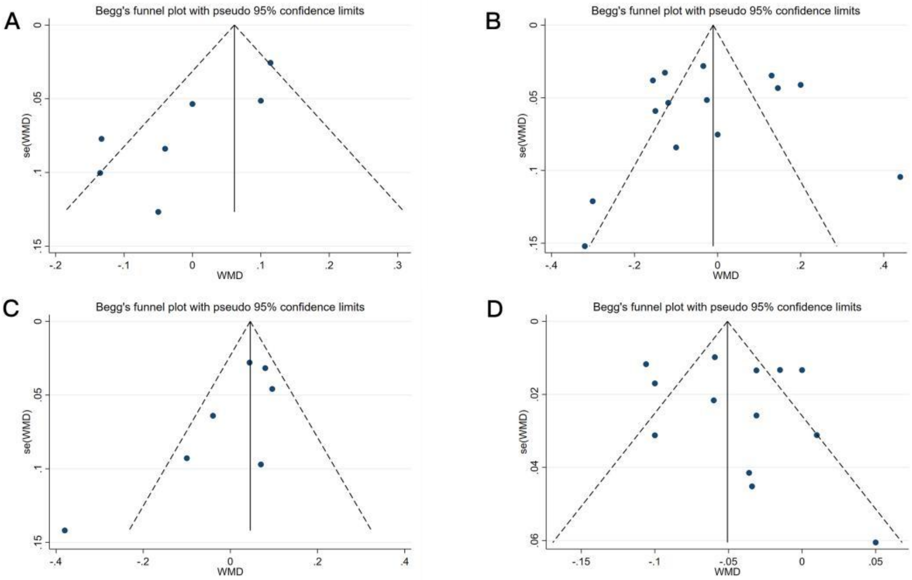

efigure 6 (A) Funnel Plots showing association between triglyceride and AF; (B) Funnel Plots showing association between total cholesterol and AF; (C) Funnel Plots showing association between low-density lipoprotein and AF; (D) Funnel Plots showing association between high-density lipoprotein and AF; AF: atrial fibrillation.

etable 1: Characteristics of Studies Included in the Meta-analysis of AF associated with Obesity

Study	Publish Year	country	Sex	Source	Mean Follow-up (y)	Mean age (y)	Exposure confirmation	Outcome Assessment	No. of Cases	No of subjects	Adjustment Variables	NOS
Denas, G[1]	2021	Italy	M,W	Population-based	1	75	Measurement	Medical records	379	14987	age, sex, number of visits, number of comorbidities，BMI，Mitral valve disease，Chronic kidney disease，Prior stroke/TIA	7
Singleton, M. J[2]	2021	US	M,W	Population-based	13.8	60	Measurement	Medical records/Self-reported	970	6739	age, sex, and race/ethnicity， current smoking, diabetes mellitus, systolic blood pressure, antihypertensive Medication use, left ventricular hypertrophy by ECG, income, education, and self-reported physical activity.	8
Tsai, S. Y[3]	2021	Taiwan	M,W	Population-based	13.7	44.1	Measurement/Self-reported	Record linkage	148	5742	sex and age，current smoking status, alcohol use, and regular exercise，marital status, education level, and average monthly income	8
Johansson, C[4]	2020	Sweden	M,W	Population-based	15	46.3	Measured/Self-reported	Swedish National Patient Registry	5154	108417	age, cardiovascular risk factors (cholesterol as a binary variable, using the 5.5 mmol/L as cut-off, education level, smoking habits, history of myocardial infarction, hypertension, diabetes, alcohol use, and physical activity)	8
Kamil-Rosenberg, S[5]	2020	US	M,W	Middle-aged and older us veterans	10.7	58.7	Measured/Self-reported /ICD coding	Record linkage/ ICD coding	2155	16397	Null	5
Younis, A[6]	2020	Israel	M,W	Self-referred subjects	7.5	49	Measured/Self-reported	Medical records	463	20410	age, gender, DM and IHD	6

Ball, J[7]	2018	Norway	M,W	Population-based	15.7	45.5	Measured/Self-reported	Record linkage and end point adjudication	1729	24799	Age， systolic blood pressure; total cholesterol, HDL cholesterol, and triglyceride levels; current daily smoking; physical activity levels; CVD (myocardial infarction, angina, and stroke); concurrent diabetes mellitus; and current antihypertensive medications.	9
Crump, C[8]	2018	Sweden	M	Swedish military conscripts	28.2	47.4	Measured	ICD coding	23600	1547478	Age,year of the military conscription examination,height, weight, aerobic fitness, muscular strength, education, neighborhood SES, and family history of AF,hypertension, diabetes mellitus, and ischemic heart disease	9
Di Benedetto, L[9]	2018	UK	M,W	Population-based	17.1	58.6	Measured/Self-reported	Medical records/Self-reported/ICD coding	2155	25639	age and sex,BMI,Physical activity ,Alcohol drinking ,Social class ,Education level ,smoking status	9
Foy, A. J[10]	2018	US	M,W	Privately insured cohort of middle-aged adults	8	43.8	database	ICD coding	1511	67278	age, gender, hypertension, and diabetes,	7
Garnvik, L. E[11]	2018	Norway	M,W	Population-based	8.1	51.5	Measured/Self-reported	Medical records/ICD coding	1459	43602	sex and age,current smoking, alcohol use, self-reported CVD and occupational status,hypertension and diabetes， physical activity	8
Persson, C. E[12]	2017	Sweden	W	Swedish medical birth registry	16.6	28.3	Measured	the Patient Registry/ICD coding	6993	1522329	age, baseline diabetes, hypertension, heart failure, smoking, pregnancy year, previous parity, educational level and heart failure during follow-up	9

Kokubo, Y[13]	2017	Japan	M,W	Population-based	13.8	58.95	Measured/Self-reported	Medical records	311	6898	age,sex,SBP and BMI categories, antihypertensive drug use, current smoking, excessive drinking, non-HDL-C, CAD, arrhythmia (other than AF), and cardiac murmur	8
O'Neal, W. T[14]	2017	US	M,W	Population-based	9.4	63	Measured/Self-reported	Medical records/Self-reported	997	13688	age,sex,income,smoking, diabetes, hypertension, obesity, exercise, dyslipidemia, left ventricular hypertrophy, and cardiovascular disease	7
Trevisan, C[15]	2017	Italy	M,W	Population-based	4.4	74.3	Measured/Self-reported	Medical records/ICD coding	115	1764	age and gender， diabetes， hypertension， cardiovascular diseases， educational level ， physical activity， smoking habits， number of drugs taken	7
Diouf, I[16]	2016	Australia	M,W	Population-based	5	56.6	Measured/Self-reported	Minnesota coding	53	5422	age, gender, BMI, smoking status, usual number of alcoholic drinks, physical activity and level of education	7
Mazzone, C[17]	2015	Italy	M,W	Patients	2.4	69	Database	Medical records/ICD coding	3379	16929	Null	4
Nyström, P. K[18]	2015	Sweden	M,W	Population-based	13.6	60	Measured/Self-reported	Medical records/ICD coding	285	4021	age,hypertension, elevated fasting glucose, sex, birth country, smoking status, alcohol intake, regular moderate-intensity exercise and history of MI	9
Sciacqua, A[19]	2015	Italy	M,W	Outpatient	4.4	60.7	Measured	Medical records/Self-reported	546	3549	age, gender, glucose, LDL-cholesterol, smoking, body mass index (BMI), and systolic blood pressure (BP), CHADS2,CHA2DS2-VASc	6
Vermond, R. A[20]	2015	Holland	M,W	Population-based	9.7	49	Measured/Self-reported	Medical records/ICD coding	265	8265	age,sex,heart failure,antihypertensive drug use, diabetes,previous stroke, previous myocardial infarction, peripheral artery disease, and NT-proBNP	8
Vermond, R.	2014	US	W	Postmenopau	11.5	63.4	Measured/S	Medical	9792	81317	age, race/ethnicity, education, BMI, HTN,	8

A[21]				sal women			elf-reported	records/ICD coding			diabetes, hyperlipidemia, coronary artery disease(CAD),CHF, PAD, smoking, and physical activity	
Chuang, S. Y[22]	2014	Taiwan	M,W	Population-b ased	9.16	72.4	Measured	Medical records/ICD coding	90	1485	Age, elevated systolic blood pressure, smoking and high uric acid,diabetes,smoking status	7
Schmidt, M[23]	2014	Denmar k	M	Young population-b ased	26	19	Measured/S elf-reported	Medical records	357	12850	years of education and height	7
Karasoy, D[24]	2013	Denmar k	W	Women without prior af who gave birth	4.6	30.6	Measured/S elf-reported	Medical records/ICD coding	110	271203	age and year of inclusion， Body mass index, age, hyperthyroidism, and previous use of beta-blockers	8
Suzuki, S[25]	2013	Japan	M,W	Patients attending the cardiovascul ar institute	7	61	Measured/S elf-reported	Medical records	2296	17517	atrial fibrillation and sex	5
Grundvold, I[26]	2012	Norway	M	Population-b ased	35	50	Measured/S elf-reported	Medical records/ICD coding	270	2014	age, systolic blood pressure, current smoking, total cholesterol, and blood glucose	8
Lipworth, L[27]	2012	US	M,W	Population-b ased	5.7	72	Self-reporte d	Medical records/ICD coding	1062	8836	race and gender	6
Chamberlain , A. M[28]	2011	US	M,W	Community-based	10	55	Measured/S elf-reported	Medical records/ICD coding	515	14546	gender, race, and continuous age	8
Smith, J. G[29]	2010	Sweden	M,W	Population-b ased	11.2	58	Measured/S elf-reported	Medical records/ICD coding	312	30447	age	8
Tedrow, U. B[30]	2010	US	W	Population-b ased	12.9	54.6	Measured/S elf-reported	Medical records	834	34309	Age，ethnicity, hypertension，hypercholesterolemia，diabetes, alcohol consumption，smoking，physical activity	9
Nichols, G.	2009	US	M,W	Kaiser	7.2	58.4	Measured/S	Medical	1059	34744	Age and sex，	7

A[31]				permanente northwest			elf-reported	records/ICD coding				
Watanabe, H[32]	2009	Japan	M,W	Community-based	4.5	59.2	Measured/Self-reported	Medical records	265	28449	Age and sex，	6
Umetani, K[33]	2007	Japan	M,W	Patients	1.7	63	Measured/Self-reported	Medical records	32	592	Null	4
Frost, L[34]	2005	Denmark	M,W	Population-based	5.7	56	Measured/Self-reported	Medical records/ICD coding	553	47589	age, systolic pressure, treatment for hypertension, together with information of body height and body mass index	8
Wang, T. J[35]	2004	US	M,W	Population-based	13.7	57	Measured/Self-reported	Medical records	526	5282	age, systolic blood pressure, use of antihypertensive therapy, diabetes mellitus, electrocardiographic left ventricular hypertrophy, history of MI or congestive heart failure	9
Wilhelmsen, L[36]	2001	Sweden	M	Population-based	25.2	51	Measured/Self-reported	Minnesota code/ICD coding/Medical records	754	7495	age,	7
Krahn, A. D[37]	1995	Canada	M	Male air crew recruits	38.7	31	Measured/Self-reported	Medical records	299	3983	age, myocardial infarction, stable or unstable angina, hyper-tension, congestive heart failure, stroke, smoking, ST- T wave abnormalities	7

Abbreviations: M: Men; W: Women; NOS: Newcastle-Ottawa score; AF:atrial fibrillation; DM:diabetes mellitus ;BMI:body mass index ; LDL:low-density lipoprotein; HDL:high-density lipoprotein;ICD：International Classification of Disease

etable 2: Characteristics of Studies Included in the Meta-analysis of AF associated with Per 5kg/m^2 Increase in BMI

Study	Publish Year	country	Sex	Source	Mean Follow-up (y)	Mean age (y)	Exposure confirmation	Outcome Assessment	No. of Cases	No of subjects	Adjustment Variables	NOS
Matsumoto, K[1]	2021	US	M,W	Older population-based	9.5	70.5	Measured/Self-reported	Medical records	83	769	age, sex, race, and hypertension，number of antihypertensive drugs	8
Singleton, M. J[2]	2021	US	M,W	Population-based	13.8	60	Measurement	Medical records/Self-reported	970	6739	age, sex, and race/ethnicity， current smoking, diabetes mellitus, systolic blood pressure, antihypertensive Medication use, left ventricular hypertrophy by ECG, income, education, and self-reported physical activity.	8
Zia, I[3]	2021	Swedish	M,W	Population-based	18.9	58	Measured/Self-reported	Record linkage	4317	25961	age, use of antihypertensive medication, lipid-lowering medication, systolic blood pressure, smoking, low physical activity, Apo-A and Apo-B blood levels and diabetes，alcohol consumption, low education, marital status and immigrant status	9
Johansson, C[4]	2020	Sweden	M,W	Population-based	15	46.3	Measured/Self-reported	Swedish National Patient Registry	5154	108417	age, cardiovascular risk factors (cholesterol as a binary variable, using the 5.5 mmol/L as cut-off, education level, smoking habits, history of myocardial infarction, hypertension, diabetes, alcohol use, and physical activity)	8
Schrage, B[5]	2020	Europe	M,W	Population-based	13.8	50.5	Record linkage/Measured/Self-reported	Measured/Self-reported/ICD coding	2959	58693	Not known	7
Berkovitch, A[6]	2019	Israel	M,W	self-referred men	7.5	49	Measured/Self-reported	Medical records	563	21118	previous cerebrovascular event, diabetes mellitus and cholesterol levels	5

				and women								
Aronson, D[7]	2018	Israel	M,W	Population-based	10	62	Measured/ Medical records	MHS'scentral databases and cardiovascular diseases registrar	5660	96778	age, sex, BMI, history of treated hypertension, SBP ≥160 mm Hg, chronic lung disease, history of myocardial infarction, history of PAD, heart failure and history of an inflammatory disease	9
Crump, C[8]	2018	Sweden	M	Swedish military conscripts	28.2	47.4	Measured	ICD coding	23600	1547478	Age,year of the military conscription examination,height, weight, aerobic fitness, muscular strength, education, neighborhood SES, and family history of AF,hypertension, diabetes mellitus, and ischemic heart disease	9
Persson，C. E[9]	2017	Sweden	W	Swedish Medical Birth Registry	16.6	28.3	Measured	the Patient Registry/ICD coding	6993	1522329	age, baseline diabetes, hypertension, heart failure, smoking, pregnancy year, previous parity, educational level and heart failure during follow-up	9
Ding, L[10]	2017	China	M,W	population-based	2.6	56.69	Measured	Medical records	134	33186	age and sex，history of CHD,hypertension, SBP, DBP, VVV in SBP, VVV in DBP, left high-amplitude R waves and premature beats	7
Magnussen, C[11]	2017	UK	M,W	population-based	12.6	49.6	Measured	Medical records/ICD coding	4261	79793	Age，body mass index, systolic blood pres- sure, diabetes mellitus, daily smok- ing, antihypertensive medication, and total cholesterol	8
Trevisan, C[12]	2017	Italy	M,W	Population-based	4.4	74.3	Measured/Self-reported	Medical records/ICD coding	115	1764	age and gender，diabetes，hypertension，cardiovascular diseases，educational level，physical activity，smoking habits，number of drugs taken	7

Karas, M. G[13]	2016	US	M,W	community-based	13	72.4	Measured/Self-reported	Medical records/Self-reported	1050	4276	age, sex, and race，the confounders smoking status, physical activity level, alcohol consumption, estrogen therapy use, and serum creatinine level，hypertension and dysglycemia measures, lipid fractions, and C-reactive protein concentration	8
Dewland, T. A[14]	2015	US	M,W	Population-based	10.9	73	Measured/Self-reported	Medical records/ICD coding	721	3075	age, sex, and race，study site.diabetes，hypertension，BMI	9
Käräjämäki, A. J[15]	2015	Finland	M,W	Middle-Aged Population	16.3	51.3	Measured/Self-reported	Medical records/ICD coding	94	958	age, sex, study group, diabetes, BMI, waist circumference, alcohol consumption, smoking,systolic blood pressure, quick index, left ventricular mass index, left atrial diameter, coronary artery disease	9
Kokubo, Y[16]	2015	Japan	M,W	Population-based	12.8	56.4	Measured	Medical records/Minnesota Code	253	6906	sex and age,BMI,BP,hypercholesterolemia, DM, and current smoking and drinking.cohort groups, chronic kidney disease, and histories of stroke, coronary heart disease, chronic heart failure, and premature contractions.	9
Nyström, P. K[17]	2015	Sweden	M,W	Population-based	13.6	60	Measured/Self-reported	Medical records/ICD coding	285	4021	age,hypertension, elevated fasting glucose, sex, birth country, smoking status, alcohol intake, regular moderate-intensity exercise and history of MI	9
Park, H. C[18]	2015	Korea	M,W	Population-based	4	60.2	Measured/Self-reported	Medical records	54	4053	Not known	6
Sciacqua, A[19]	2015	Italy	M,W	Outpatient	4.4	60.7	Measured	Medical records/Self-reported	546	3549	age, gender, glucose, LDL-cholesterol, smoking, body mass index (BMI), and systolic blood pressure (BP), CHADS2,CHA2DS2-VASc	6
Vermond, R. A[20]	2015	Holland	M,W	Population-based	9.7	49	Measured/Self-reported	Medical records/ICD coding	265	8265	age , sex ,heart failure, antihypertensive drug use, diabetes,previous stroke, previous myocardial infarction, peripheral artery disease, and NT-proBNP	8

Knuiman, M[21]	2014	Australia	M,W	Population-based	15	52	Measured/Self-reported	Medical records/ICD coding	343	4267	Sex, age, height, hypertension treatment, and BMI	9
Mandalenakis, Z[22]	2014	Sweden	M	Population-based	16	70	Measured/Self-reported	Medical records/ICD coding	90	423	age,systolic and diastolic BP, serum triglycerides, smoking, serum cholesterol, fasting glucose, body mass index, heart rate, and ANP 2, 3, and 4.	9
Schmidt, M[23]	2014	Denmark	M	Young Population-based	26	19	Measured/Self-reported	Medical records	357	12850	years of education and height	7
Grundvold, I.[24]	2013	Oslo	M	Population-based	30	71	Measured	Medical records/ICD coding	270	2341	age, systolic BP , physical fitness, and relative heart volume	7
Nyrnes, A[25]	2013	Norway	M,W	Population-based	11.1	46	Measured/Self-reported	Medical records/ICD coding	361	22815	age, BMI, height, SBP, DBP, heart rate, total cholesterol, HDLcholesterol, current smoking, physical activity, alcohol and coffee consumption, prevalent CHD, diabetes, hypertension, anti-hypertensive treatment and palpitations.	9
Perez, M. V[26]	2013	US	W	postmenopausal women	9.8	63	Measured/Self-reported	Medical records/ICD coding	8252	81892	Age, hypertension, obesity, diabetes, myocardial infarction and heart failure	8
Casaclang-Verzosa, G[27]	2010	US	M,W	High-Risk Elderly, SAFHIRE	1.7	74	Measured/Self-reported	Medical records	34	800	Age，and sex，	5
Tedrow, U. B[28]	2010	US	W	Population-based	12.9	54.6	Measured/Self-reported	Medical records	834	34309	Age，ethnicity, hypertension，hypercholesterolemia，diabetes, alcohol consumption，smoking，physical activity	9
Frost, L[29]	2005	Denmark	M,W	Populati	5.7	56	Measured/S	Medical	553	47589	age, systolic pressure, treatment for hypertension, together	8

				on-based			elf-reported	records/ICD coding			with information of body height and body mass index	
Wang, T. J[30]	2004	US	M,W	Populati on-based	13.7	57	Measured/S elf-reported	Medical records	526	5282	age, systolic blood pressure, use of antihypertensive therapy, diabetes mellitus, electrocardiographic left ventricular hypertrophy, history of MI or congestive heart failure	9
Wilhelmsen , L[31]	2001	Sweden	M	Populati on-based	25.2	51	Measured/S elf-reported	Minnesota code/ICD coding/Medical records	754	7495	age,	7

Abbreviations: M: Men; W: Women; Nos: Newcastle-Ottawa score; AF:atrial fibrillation; DM:diabetes mellitus ;BMI:body mass index ; LDL:low-density lipoprotein; HDL:high-density lipoprotein;ICD：International Classification of Disease

etable 3: Characteristics of Studies Included in the Meta-analysis of AF associated with BMI

Study	Publish Year	country	Sex	Source	Mean Follow-up (y)	Mean age (y)	Exposure confirmation	Outcome Assessment	No. of Cases	No of subjects	NOS
Denas, G[1]	2021	Italy	M,W	Population-based	1	75	Measurement	Medical records	379	14987	7
Espnes, H[2]	2021	Norway	M,W	Population-based	17.6	46	Measurement , Self-reported	Medical records	1517	24804	8
Bundy, J. D[3]	2020	US	W，M	Population-based	11.4	61.3	Measured/Self-reported	Medical records/Self-reported	436	3534	8
Persson, A. P[4]	2020	Sweden	W，M	Population-based	17	65	Measured/Self-reported	Based on ICD code	80	377	8
Andersen, K[5]	2018	Sweden	M	Young men	26.3	18.2	Measured	Based on ICD code	9753	1153151	7
Garg, P. K[6]	2018	US	W，M	Non-hispanic black and white adults	9.4	63	Measured/Self-reported	Medical records/Self-reported	725	9576	7
Tikhonoff, V[7]	2018	Belgium	W，M	Population-based	14	43.1	Measured/Self-reported	Medical records	143	3956	8
Ding, L[8]	2017	China	M,W	Population-based	2.6	56.69	Measured	Medical records	134	33186	7

Kokubo, Y[9]	2017	Japan	M,W	Population-based	13.8	58.95	Measured/Self-reported	Medical records	311	6898	8
Perkiömäki, J. S[10]	2017	Finland	M,W	Population-based	16.4	51.95	Measured	Based on ICD code	91	903	9
Raman, D[11]	2017	US	M	Older community-dwelling men	8	75.8	Measured/Self-reported	Medical records	269	2350	6
Trevisan, C[12]	2017	Italy	M,W	Population-based	4.4	74.3	Measured/Self-reported	Medical records/ICD coding	115	1764	7
Ermakov, S[13]	2016	US	W	Postmenopausal women	11.1	65.7	Measured/Self-reported	Medical records/ICD coding	892	4937	8
Lim, C. W[14]	2016	Malaysia	M,W	Community-based	15	52.6	Measured/Self-reported	Medical records	53	10805	8
Nyström, P. K[15]	2015	Sweden	M,W	Population-based	13.6	60	Measured/Self-reported	Medical records/ICD coding	285	4021	9
Park, H. C[16]	2015	Korea	M,W	Population-based	4	60.2	Measured/Self-reported	Medical records	54	4053	6
Sciacqua, A[17]	2015	Italy	M,W	Outpatient	4.4	60.7	Measured	Medical records/Self-reported	546	3549	6
Vermond, R. A[18]	2015	Holland	M,W	Population-based	9.7	49	Measured/Self-reported	Medical records/ICD coding	265	8265	8
Chuang, S. Y[19]	2014	Taiwan	M,W	Population-based	9.16	72.4	Measured	Medical records/ICD coding	90	1485	7
McManus, D. D[20]	2014	US	M,W	Community-based	5.4	66.3	Measured	Medical records	107	2445	6

Agarwal, S. K[21]	2013	US	M,W	Population-based	18.1	55.3	Measured/Self-reported	Medical records/ICD coding	1438	12071	6
Karppi, J[22]	2013	Finland	M,W	Population-based	2.8	71	Measured/Self-reported	Medical records/ICD coding	105	2340	7
Suzuki, S[23]	2013	Japan	M,W	Patients attending the cardiovascular institute	7	61	Measured/Self-reported	Medical records	2296	17517	5
Grundvold, I[24]	2012	Norway	M	Population-based	35	50	Measured/Self-reported	Medical records/ICD coding	270	2014	8
Ninios, I[25]	2010	Greece	M,W	Population-based	2	72.5	Measured/Self-reported	Medical records	36	720	4
Patton, K. K[26]	2009	US	M,W	Community-based	10	73.6	Measured/Self-reported	Medical records/ICD coding	1126	5021	7
Ravn, L. S[27]	2008	Denmark	M,W	Population-based	26	62	Measured/Self-reported	Medical records/ICD coding	968	9235	6
Watanabe, H[28]	2006	Japan	M,W	Community-based	10	61.3	Measured/Self-reported	Medical records	873	63386	6

Abbreviations: M: Men; W: Women; Nos: Newcastle-Ottawa score; AF:atrial fibrillation; DM:diabetes mellitus ;BMI:body mass index ;ICD：International Classification of Disease

etable 4: Characteristics of Studies Included in the Meta-analysis of AF associated with Smoking

Study	Publish Year	country	Sex	Source	Mean Follow-up (y)	Mean age (y)	Exposure confirmation	Outcome Assessment	No of Cases	No of subjects	Adjustment Variables	NOS
Banks, E[1]	2019	Australia	M,W	Aged ≥ 45 years general population	7.2	62.5	Self-reported	Medical records/Record linkage	5238	188167	age, sex, urban/rural residence, alcohol consumption, income and education	7
Zuo, H[2]	2018	Norway	M,W	Community-based	11	50	Measured/Self-reported	Medical records/ICD coding	538	6682	gender, age, body mass index, hypertension, physical activity and education	9
Kokubo, Y[3]	2017	Japan	M,W	Population-based	13.8	58.95	Measured/Self-reported	Medical records	311	6898	age,sex,SBP and BMI categories, antihypertensive drug use, current smoking, excessive drinking, non-HDL-C, CAD, arrhythmia (other than AF), and cardiac murmur	8
Diouf, I[4]	2016	Australia	M,W	Population-based	5	56.6	Measured/Self-reported	Minnesota coding	53	5422	age, gender, BMI, smoking status, usual number of alcoholic drinks, physical activity and level of education	7
Lim, C. W[5]	2016	Malaysia	M,W	Community-based	15	52.6	Measured/Self-reported	Medical records	53	10805	age	8
Dewland, T. A[6]	2015	US	M,W	Population-based	10.9	73	Measured/Self-reported	Medical records/ICD coding	721	3075	age, sex, and race， study site.diabetes，hypertension，BMI	9
Suzuki, S[7]	2015	Japan	M,W	Patients of the cardiovascular institute	2	56.7	Measured/Self-reported	Medical records	190	15221	age, sex, diabetes ， hypertension ，BMI,Dyslipidemia,Heart failure ,	8
Tiwari, S[8]	2015	Norway	M,W	Caucasians	16	62.6	Measured/Self-reported	Medical	462	2406	Not known	6

				population-based				records/ICD coding				
Adamsson, E. S[9]	2014	Sweden	M,W	Population-based	15.3	57.4	Measured/Self-reported	Medical records/ICD coding	353	4836	age and sex， smoking, diabetes, waist circumference, systolic blood pressure, anti-hypertensive medication, LDL, HDL, education, physical activity and CRP	8
Chrispin, J[10]	2014	US	M,W	Population-based	6.9	65.5	Measured/Self-reported	Medical records/ICD coding	214	4942	age, sex, race, body mass index, cigarette smoking status, systolic blood pressure, diabetes, total cholesterol, high-density lipoprotein cholesterol, and use of digitalis, antiarrhythmic, antihypertensive, and lipid medications	7
Chuang, S. Y[11]	2014	Taiwan	M,W	Population-based	9.16	72.4	Measured	Medical records/ICD coding	90	1485	Age, elevated systolic blood pressure, smoking and high uric acid,diabetes,smoking status	7
Knuiman, M[12]	2014	Australia	M,W	Population-based	15	52	Measured/Self-reported	Medical records/ICD coding	343	4267	Sex, age, height, hypertension treatment, and BMI	9
Perez, M. V[13]	2013	US	W	Postmenopausal women	9.8	63	Measured/Self-reported	Medical records/ICD coding	8252	81892	Age, hypertension, obesity, diabetes, myocardial infarction and heart failure	8
Chamberlain, A. M[14]	2011	US	M,W	Community-based	13.1	54.1	Measured/Self-reported	Medical records/ICD coding	876	15329	age, sex, race, ARIC field center, education, BMI, alcohol drinking status , sports index , diabetes, and hypertension	9
Ninios, I[15]	2010	Greece	M,W	Population-based	2	72.5	Measured/Self-reported	Medical records	36	720	Not known	4
Rosengren,	2009	Sweden	M	Population-based	25	51.5	Measured/Self-reported	Medical	1253	6903	age,	8

A[16]								records/ICD coding				
Heeringa, J[17]	2008	Holland	M,W	Population-based	7.2	68.1	Measured/Self-reported	Medical records	371	5668	age, gender, body mass index, hypertension, systolic blood pressure, serum cholesterol level, diabetes mellitus, left ventricular hypertrophy on the electrocardiogram, prevalent and incident myocardial infarction, prevalent heart failure, and the use of pulmonary medication.	7
Frost, L[18]	2005	Denmark	M,W	Population-based	5.7	56	Measured/Self-reported	Medical records/ICD coding	553	47589	age, systolic pressure, treatment for hypertension, together with information of body height and body mass index	8

Abbreviations: M: Men; W: Women; Nos: Newcastle-Ottawa score; AF:atrial fibrillation; DM:diabetes mellitus ;BMI:body mass index;ICD：International Classification of Disease

etable 5: Characteristics of Studies Included in the Meta-analysis of AF associated with Diabetes

Study	Publish Year	country	Sex	Source	Mean Follow-up (y)	Mean age (y)	Exposure confirmation	Outcome Assessment	No. of Cases	No of subjects	Adjustment Variables	NOS
Chao, T. F[1]	2021	Taiwan	M, W	Population-based	16	53	Physician-diagnosed	Medical records	438930	7220654	age, male sex, and important comorbidities (hypertension, heart failure, coronary artery disease, end- stage renal disease, and alcoholism)	8
Denas, G[2]	2021	Italy	M, W	Population-based	1	75	Physician-diagnosed/screen-detected	Medical records	379	14987	age (per each year increment), male sex , over-<br>weight (BMI ≥ 28.4 kg/m2), mitral valve disease, congestive heart failure, and the number annual of visits , systolic blood pressure, mitral valve disease, stroke/TIA, and chronic kidney disease	7
Espnes, H[3]	2021	Norway	M, W	Population-based	17.6	46	Measurement , Self-reported	Medical records	2018	24804	age, body mass index, total cholesterol, current smoking, leisure time physical activity , and history of myocardial infarction, angina pectoris, stroke, and diabetes mellitus	8
Himmelreich, J[4]	2021	Netherlands	M, W	Community-based	5	65.5	Measurement/Medical records/database	Measurement/Medical records/database	5264	111475	CHARGE- AF, CHA2DS2- V ASc and age alone	6
Viktor Lind[5]	2021	Swedish	M, W	Population-based	19.1	51.5	Physician-diagnosed/screen-detected/index	Medical records/Record	28233	294057	sex, age, total cholesterol, triglyc-erides and socioeconomic status, BMI, smoking,	8

							examination	linkage			alcohol drinking and physical activity.	
Matsumoto, K[6]	2021	Northern Manhattan	M, W	Older population-based	9.5	70.5	Measured/Self-reported	Medical records	83	769	age, sex, race, and hypertension，number of antihypertensive drugs	7
Schmidt, C[7]	2021	Germany	M, W	Patients	3	63	Measured	Medical records	34	305	age,gender and BMI	5
Schrage, B[8]	2020	Europe	M, W	Population-based	13.8	50.5	Record linkage/Measured/Self-reported	Measured/Self-reported/ICD coding	2959	58693	Not known	7
Staerk, L[9]	2020	US	M, W	Population-based	10	61	Measured/Self-reported /Medical records	Medical records	822	7586	race ,age , height , weight, systolic blood pressure, diastolic blood pressure, current smoking, antihypertensive treatment, diabetes mellitus, and adjudicated heart failure and myocardial infarction	9
Younis, A[10]	2020	Israel	M, W	Self-referred subjects	7.5	49	Measured/Self-reported	Medical records	463	20410	age, gender, DM and IHD	6
Li, Y. G[11]	2019	China Yunnan	M, W	Population-based	4.1	47	Yunnan Medical Insurance Database	Medical records/ICD code	921	471446	structural heart disease (SHD), heart failure (HF), age≥75years, coronary artery disease (CAD), hyperthyroidism, COPD, and hypertension	7
Austin, T. R[12]	2018	US	W，M	Population-based	8.5	55.2	Measured/Self-reported	Medical records/ICD code	242	5240	age, sex,weight, antihypertensive medication use, higher SBP, history of MI, lower FEV1 and eGFR (<60mL/min per m2), and current smoking	8
Crump, C[13]	2018	Sweden	M	Swedish military conscripts	28.2	47.4	Measured	ICD coding	23600	1547478	Age,year of the military conscription examination,height, weight, aerobic fitness, muscular strength, education, neighborhood	9

											SES, and family history of AF,hypertension, diabetes mellitus, and ischemic heart disease	
Foy, A. J[14]	2018	US	M, W	Privately insured cohort of middle-aged adults	8	43.8	database	ICD coding	1511	67278	Not known	4
Tikhonoff, V[15]	2018	Belgium	W，M	Population-based	14	43.1	Measured/Self-reported	Medical records	143	3956	sex, age, body mass index, serum cholesterol, tobacco and alcohol use, history of cardiovascular disease and diabetes mellitus and antihypertensive drug treatment	8
Ding, L[16]	2017	China	M, W	Population-based	2.6	56.69	Measured	Medical records	134	33186	age and sex，history of CHD,hypertension, SBP, DBP, VVV in SBP, VVV in DBP, left high-amplitude R waves and premature beats	7
Gillott, R. G[17]	2017	UK	M, W	Population-based	2	44.12	the GP database	QOF registry data	5304	277218	age,sex,hypertension, heart failure, diabetes, and IHD.	7
Kokubo, Y[18]	2017	Japan	M, W	Population-based	13.8	58.95	Measured/Self-reported	Medical records	311	6898	age,sex,SBP and BMI categories, antihypertensive drug use, current smoking, excessive drinking, non-HDL-C, CAD, arrhythmia (other than AF), and cardiac murmur	8
Magnussen, C[19]	2017	UK	M, W	Population-based	12.6	49.6	Measured	Medical records/ICD coding	4261	79793	Age，body mass index, systolic blood pressure, diabetes mellitus, daily smoking, antihypertensive medication, and total cholesterol	8

O'Neal, W. T[20]	2017	US	M, W	Population-based	9.4	63	Measured/Self-reported	Medical records/Self-reported	997	13688	age,sex,income,smoking, diabetes, hypertension, obesity, exercise, dyslipidemia, left ventricular hypertrophy, and cardiovascular disease	7
Raman, D[21]	2017	US	M	Older community-dwelling men	8	75.8	Measured/Self-reported	Medical records	269	2350	age,race, body mass index, waist circumference, cardiac medications, comorbid diseases, alcohol use, and study site.	6
Trevisan, C[22]	2017	Italy	M, W	Population-based	4.4	74.3	Measured/Self-reported	Medical records/ICD coding	115	1764	age and gender，diabetes，hypertension，cardiovascular diseases，educational level ，physical activity，smoking habits， number of drugs taken	6
Diouf, I[23]	2016	Australia	M, W	Population-based	5	56.6	Measured/Self-reported	Minnesota coding	53	5422	age, gender, BMI, smoking status, usual number of alcoholic drinks, physical activity and level of education	7
Lim, C. W[24]	2016	Malaysia	M, W	Community-based	15	52.6	Measured/Self-reported	Medical records	53	10805	age	8
Dewland, T. A[25]	2015	US	M, W	Population-based	10.9	73	Measured/Self-reported	Medical records/ICD coding	721	3075	age, sex, and race， study site.diabetes，hypertension，BMI	9
Käräjämäki, A. J[26]	2015	Finland	M, W	Middle-aged population	16.3	51.3	Measured/Self-reported	Medical records/ICD coding	94	958	age, sex, study group, diabetes, BMI, waist circumference, alcohol consumption, smoking,systolic blood pressure, quick index, left ventricular mass index, left atrial diameter, coronary artery disease	9

Mazzone, C[27]	2015	Italy	M, W	Patients	2.4	69	Database	Medical records/ICD coding	3379	16929	Not known	4
Sciacqua, A[28]	2015	Italy	M, W	Outpatient	4.4	60.7	Measured	Medical records/Self-reported	546	3549	age, gender, glucose, LDL-cholesterol, smoking, body mass index (BMI), and systolic blood pressure (BP), CHADS2,CHA2DS2-VASc	6
Vermond, R. A[29]	2015	Holland	M, W	Population-based	9.7	49	Measured/Self-reported	Medical records/ICD coding	265	8265	age , sex ,heart failure, antihypertensive drug use, diabetes,previous stroke, previous myocardial infarction, peripheral artery disease, and NT-proBNP	8
Chrispin, J[30]	2014	US	M, W	Population-based	6.9	65.5	Measured/Self-reported	Medical records/ICD coding	214	4942	age, sex, race, body mass index, cigarette smoking status, systolic blood pressure, diabetes, total cholesterol, high-density lipoprotein cholesterol, and use of digitalis, antiarrhythmic, antihypertensive, and lipid medications	7
Frost, L[31]	2014	Denmark	M, W	Population-based	13.5	56.1	Measured/Self-reported	Medical records/ICD coding	2581	55273	age, sex,smoking status, educational level, and physical activity.hypertension, diabetes mellitus, hypercholesterolemia, ischemic heart disease,congestive heart failure, and valvular heart disease	9
Knuiman, M[32]	2014	Australia	M, W	Population-based	15	52	Measured/Self-reported	Medical records/ICD coding	343	4267	Sex, age, height, hypertension treatment, and BMI	9
Mora, S[33]	2014	US	W	Healthy female	16.4	55.65	Measured/Self-reported	Medical records	795	23738	age, race, trial treatment assignment,	8

				healthcare professionals							menopausal status, HRT, smoking, income, education, blood pressure, antihypertensive medication use, diabetes, exercise, alcohol use, and BMI,inflammatory/ endothelial function markers,hemoglobin A1c, and creatinine	
Karppi, J[34]	2013	Finland	M, W	Population-based	2.8	71	Measured/Self-reported	Medical records/ICD coding	105	2340	Not known	7
Perez, M. V[35]	2013	US	W	Postmenopausal women	9.8	63	Measured/Self-reported	Medical records/ICD coding	8252	81892	Age, hypertension, obesity, diabetes, myocardial infarction and heart failure	8
Suzuki, S[36]	2013	Japan	M, W	Patients attending the cardiovascular institute	7	61	Measured/Self-reported	Medical records	2296	17517	atrial fibrillation and sex	5
Lipworth, L[37]	2012	US	M, W	Population-based	5.7	72	Self-reported	Medical records/ICD coding	1062	8836	race and gender	6
Chamberlain, A. M[38]	2011	US	M, W	Community-based	10	55	Measured/Self-reported	Medical records/ICD coding	515	14546	gender, race, and continuous age	8
Casaclang-Verzosa, G[39]	2009	US	M, W	High-risk elderly, safhire	1.7	74	Measured/Self-reported	Medical records	34	800	Age，and sex，	5
Ninios, I[40]	2010	Greece	M, W	Population-based	2	72.5	Measured/Self-reported	Medical records	36	720	Not known	4
Smith, J. G[41]	2010	Sweden	M,	Population-based	11.2	58	Measured/Self-reported	Medical	312	30447	age	8

			W					records/ICD coding				
Nichols, G. A[42]	2009	US	M, W	Kaiser permanente northwest	7.2	58.4	Measured/Self-reported	Medical records/ICD coding	1059	34744	Age and sex，	7
Patton, K. K[43]	2009	US	M, W	Community-based	10	73.6	Measured/Self-reported	Medical records/ICD coding	1126	5021	Not known	7
Rosengren, A[44]	2009	Sweden	M	Population-based	25	51.5	Measured/Self-reported	Medical records/ICD coding	1253	6903	age	8
Umetani, K[45]	2007	Japan	M, W	Patients	1.7	63	Measured/Self-reported	Medical records	32	592	Not known	4
Watanabe, H[46]	2006	Japan	M, W	Community-based	10	61.3	Measured/Self-reported	Medical records	873	63386	Not known	6
Friberg, J[47]	2003	Denmark	M, W	Population-based	10	69.5	Measured/Self-reported	Medical records	281	13391	Age and sex，arterial hypertension, systolic blood pressure on the day of examination, electrocardiographic LVH, prior myocardial infarction, diabetes mellitus, tobacco smoking, alcohol consumption, height, body weight, and BMI	7
Krahn, A. D[48]	1995	Canada	M	Male air crew recruits	38.7	31	Measured/Self-reported	Medical records	299	3983	age	6

Abbreviations: M: Men; W: Women; NOS: Newcastle-Ottawa score; AF:atrial fibrillation; DM:diabetes mellitus ;BMI:body mass index ;IHD:ischemic heart disease; LDL:low-density lipoprotein; HDL:high-density lipoprotein;ICD：International Classification of Disease

etable 6: Characteristics of Studies Included in the Meta-analysis of AF associated with Hypertension

Study	Publish Year	country	Sex	Source	Mean Follow-up (y)	Mean age (y)	Exposure confirmation	Outcome Assessment	No of Cases	No of subjects	Adjustment Variables	NOS
Chao, T. F[1]	2021	Taiwan	W，M	Population-based	16	53	Measurement	Medical record	438930	7220654	age, male sex, and important comorbidities (hypertension, heart failure, coronary artery disease, end- stage renal disease, and alcoholism)	8
Denas, G[2]	2020	Italy	M,W	Population-based	1	75	Physician-diagnosed/screen-detected	Medical records	379	14987	age (per each year increment), male sex，over-<br>weight (BMI ≥ 28.4 kg/m2), mitral valve disease, congestive heart failure, and the number annual of visits， systolic blood pressure, mitral valve disease, stroke/TIA, and chronic kidney disease	7
Espnes, H[3]	2021	Norway	M,W	Population-based	17.6	46	Measurement ，Self-reported	Medical records	2018	24804	age, body mass index, total cholesterol, current smoking, leisure time physical activity ， and history of myocardial infarction, angina pectoris, stroke, and diabetes mellitus	8
Himmelreich, J[4]	2021	Netherlands	M,W	Community-based	5	65.5	Measurement/Medical records/database	Measurement/Medical records/database	5264	111475	CHARGE- AF, CHA2DS2- V ASc and age alone	6
Matsumoto, K[5]	2021	US	M,W	Older population-based	9.5	70.5	Measured/Self-reported	Medical records	83	769	age, sex, race, and hypertension，number of antihypertensive drugs	8
Lip, G[6]	2020	Danish	M,W	All danish	5	69	Record linkage	Based on ICD	1205	249923	Null	5

				Citizens aged ≥ 65 years				code	02	5		
Niklas, A[7]	2020	Poland	M,W	Adult polish Population	1	49.2	Measured/Self-reporte d	Medical records	269	6163	Null	5
Younis, A[8]	2020	Israel	M,W	Self-referred subjects	7.5	49	Measured/Self-reporte d	Medical records	463	20410	age, gender, DM and IHD	6
Li, Y. G[9]	2019	China	M,W	Population-based	4.1	47	Yunnan Medical Insurance Database	Medical records/ICD code	921	471446	structural heart disease (SHD), heart failure (HF), age ≥ 75years, coronary artery disease (CAD), hyperthyroidism, COPD, and hypertension	7
Rattani, A[10]	2019	US	M,W	Population-based	21.4	54.1	Measured/Self-reporte d	Medical records/ICD coding	2891	14915	age, sex, and race，education, study center, height, BMI,smoking status, alcohol use, diabetes, heart failure, coronary heart disease, and stroke.	9
Aronson, D[11]	2018	Israel	M,W	Population-based	10	62	Measured/Medical records	MHS'scentral databases and cardiovascular diseases registrar	5660	96778	age,sex,BMI ,history of treated hypertension, SBP ≥160 mm Hg, chronic lung disease, history of myocardial infarction, history of PAD, heart failure and history of an inflammatory disease	9
Crump, C[12]	2018	Sweden	M	Swedish military conscripts	28.2	47.4	Measured	ICD coding	2360 0	154747 8	Age,year of the military conscription examination,height, weight, aerobic fitness, muscular strength, education, neighborhood SES, and family history of AF,hypertension, diabetes mellitus, and ischemic heart disease	9

Foy, A. J[13]	2018	US	M,W	Privately insured cohort of middle-aged adults	8	43.8	database	ICD coding	1511	67278	Not known	4
Ding, L[14]	2017	China	M,W	Population-based	2.6	56.69	Measured	Medical records	134	33186	age and sex，history of CHD,hypertension, SBP, DBP, VVV in SBP, VVV in DBP, left high-amplitude R waves and premature beats	7
Gillott, R. G[15]	2017	UK	M,W	Population-based	2	44.12	the GP database	QOF registry data	5304	277218	age, sex,hypertension, heart failure, diabetes, and IHD.	7
O'Neal, W. T[16]	2017	US	M,W	Population-based	9.4	63	Measured/Self-reported	Medical records/Self-reported	997	13688	age,sex,income,smoking, diabetes, hypertension, obesity, exercise, dyslipidemia, left ventricular hypertrophy, and cardiovascular disease	7
Perkiömäki, J. S[17]	2017	Finland	M,W	Population-based	16.4	51.95	Measured	Based on ICD code	91	903	age, sex, body mass index, height, smoking history, alanine aminotransferase, uric acid, and fasting plasma glucose	9
Raman, D[18]	2017	US	M	Older community-dwelling men	8	75.8	Measured/Self-reported	Medical records	269	2350	age,race, body mass index, waist circumference, cardiac medications, comorbid diseases, alcohol use, and study site.	6
Trevisan, C[19]	2017	Italy	M,W	Population-based	4.4	74.3	Measured/Self-reported	Medical records/ICD coding	115	1764	age and gender，diabetes，hypertension，cardiovascular diseases ， educational level ， physical activity，smoking habits，number of drugs taken	6
Diouf, I[20]	2016	Australia	M,W	Population-based	5	56.6	Measured/Self-reporte	Minnesota	53	5422	age, gender, BMI, smoking status, usual	7

							d	coding			number of alcoholic drinks, physical activity and level of education	
Lim, C. W[21]	2016	Malaysia	M,W	Community-based	15	52.6	Measured/Self-reported	Medical records	53	10805	age	8
Dewland, T. A[22]	2015	US	M,W	Population-based	10.9	73	Measured/Self-reported	Medical records/ICD coding	721	3075	age, sex, and race， study site.diabetes，hypertension，BMI	9
Khan, H[23]	2015	Finland	M	Community-based	19.5	52.6	Measured	Medical records	305	1950	Age， systolic blood pressure, history of CVD, diabetes, resting heart rate, and left ventricular hypertrophy	8
Kokubo, Y[24]	2015	Japan	M,W	Population-based	12.8	56.4	Measured	Medical records/Minnesota Code	253	6906	sex and age,BMI,BP,hypercholesterolemia, DM, and current smoking and drinking.cohort groups, chronic kidney disease, and histories of stroke, coronary heart disease, chronic heart failure, and premature contractions.	9
Mazzone, C[25]	2015	Italy	M,W	Patients	2.4	69	Database	Medical records/ICD coding	3379	16929	Not known	4
Nyström, P. K[26]	2015	Sweden	M,W	Population-based	13.6	60	Measured/Self-reported	Medical records/ICD coding	285	4021	age,hypertension, elevated fasting glucose, sex, birth country, smoking status, alcohol intake, regular moderate-intensity exercise and history of MI	9
O'Neal, W. T[27]	2015	US	M,W	Population-based	5.3	62	Measured/Self-reported	Medical records/ICD coding	182	5311	age,sex,race/ethnicity,income,and education,smoking, diabetes, body mass index, total cholesterol, HDL-cholesterol,	7

											lipid-lowering medications, aspirin use, and left ventricular hypertrophy,	
Sciacqua, A[28]	2015	Italy	M,W	Outpatient	4.4	60.7	Measured	Medical records/Self-reported	546	3549	age, gender, glucose, LDL-cholesterol, smoking, body mass index (BMI), and systolic blood pressure (BP), CHADS2,CHA2DS2-VASc	6
Torén, K[29]	2015	Sweden	M	Population-based	16.8	55.3	Measured/Self-reported	Medical records/ICD coding	436	6035	age, smoking, socioeconomic status, hypertension, body mass index and diabetes	8
Vermond, R. A[30]	2015	Netherlands	M,W	Population-based	9.7	49	Measured/Self-reported	Medical records/ICD coding	265	8265	age , sex ,heart failure, antihypertensive drug use, diabetes,previous stroke, previous myocardial infarction, peripheral artery disease, and NT-proBNP	8
Frost, L[31]	2014	Denmark	M,W	Population-based	13.5	56.1	Measured/Self-reported	Medical records/ICD coding	2581	55273	age, sex,smoking status, educational level, and physical activity.hypertension, diabetes mellitus, hypercholesterolemia, ischemic heart disease,congestive heart failure, and valvular heart disease	9
Mora, S[32]	2014	US	W	Healthy female healthcare professionals	16.4	55.65	Measured/Self-reported	Medical records	795	23738	age, race, trial treatment assignment, menopausal status, HRT, smoking, income, education, blood pressure, antihypertensive medication use, diabetes, exercise, alcohol use, and BMI,inflammatory/ endothelial function markers,hemoglobin A1c, and creatinine	8

Perez, M. V[33]	2013	US	W	Postmenopausal women	9.8	63	Measured/Self-reported	Medical records/ICD coding	8252	81892	Age, hypertension, obesity, diabetes, myocardial infarction and heart failure	8
Suzuki, S[34]	2013	Japan	M,W	Patients attending the cardiovascular institute	7	61	Measured/Self-reported	Medical records	2296	17517	atrial fibrillation and sex	5
Lipworth, L[35]	2012	US	M,W	Population-based	5.7	72	Self-reported	Medical records/ICD coding	1062	8836	race and gender	6
Ninios, I[36]	2010	Greece	M,W	Population-based	2	72.5	Measured/Self-reported	Medical records	36	720	Not known	4
Smith, J. G[37]	2010	Sweden	M,W	Population-based	11.2	58	Measured/Self-reported	Medical records/ICD coding	312	30447	age	8
Nichols, G. A[38]	2009	US	M,W	Kaiser permanente northwest	7.2	58.4	Measured/Self-reported	Medical records/ICD coding	1059	34744	Age and sex，	7
Schnabel, R. B[39]	2009	US	M,W	Community-based	10	60.9	Measured	Medical records	457	4764	Age and Sex	7
Ravn, L. S[40]	2008	Denmark	M,W	Population-based	26	62	Measured/Self-reported	Medical records/ICD coding	968	9235	Not known	6
Umetani, K[41]	2007	Japan	M,W	Patients	1.7	63	Measured/Self-reported	Medical records	32	592	Not known	4
Watanabe, H[42]	2006	Japan	M,W	Community-based	10	61.3	Measured/Self-reported	Medical records	873	63386	Not known	6

Krahn, A. D[43]	1995	Canada	M	Male air crew recruits	38.7	31	Measured/Self-reported	Medical records	299	3983	age,myocardial infarction,stable or unstable angina, hyper- tension, congestive heart failure, stroke, smoking, ST- T wave abnormalities	7
Lake, F. R[44]	1989	Australia	M,W	Community-based	15	68.5	Measured/Self-reported	Minnesota code/ICD coding/Medical records	87	1770	age, sex, history of a myocardial infarction, an abnormal electrocardiogram, angina, cholesterol level, systolic blood pressure and Quetelet's Index	7

Abbreviations: M: Men; W: Women; Nos: Newcastle-Ottawa score; AF:atrial fibrillation; DM:diabetes mellitus ;BMI:body mass index；ICD：International Classification of Disease

etable 7: Characteristics of Studies Included in the Meta-analysis of AF associated with Dyslipidemia

Study	Publish Year	country	Sex	Source	Mean Follow-up (y)	Mean age (y)	Exposure confirmation	Outcome Assessment	No. of Cases	No of subjects	Adjustment Variables	NOS
Himmelreich, J[1]	2021	Netherlands	M,W	Community-based	5	65.5	Measurement/Medical records/database	Measurement /Medical records/database	5264	111475	CHARGE- AF, CHA2DS2- V ASc and age alone	6
Matsumoto, K[2]	2021	Northern Manhattan	M,W	Older population-based	9.5	70.5	Measured/Self-reported	Medical records	83	769	age, sex, race, and hypertension，number of antihypertensive drugs	8
Schmidt, C[3]	2021	Germany	M,W	Patients	3	63	Measured	Medical records	34	305	age,gender and BMI	5
Younis, A[4]	2020	Israel	M,W	Self-referred subjects	7.5	49	Measured/Self-reported	Medical records	463	20410	age, gender, DM and IHD	6
Li, Y. G[5]	2019	China	M,W	Population-based	4.1	47	Yunnan Medical Insurance Database	Medical records/ICD	921	471446	structural heart disease (SHD), heart failure (HF), age≥75years, coronary artery disease	7

								code			(CAD), hyperthyroidism, COPD, and hypertension	
O'Neal, W. T[6]	2017	US	M,W	Population-based	9.4	63	Measured/Self-reported	Medical records/Self-reported	997	13688	age,sex,income,smoking, diabetes, hypertension, obesity, exercise, dyslipidemia, left ventricular hypertrophy, and cardiovascular disease	7
Vermond, R. A[7]	2015	Holland	M,W	Population-based	9.7	49	Measured/Self-reported	Medical records/ICD coding	265	8265	age,sex,heart failure, antihypertensive drug use, diabetes,previous stroke, previous myocardial infarction, peripheral artery disease, and NT-proBNP	8
Sciacqua, A[8]	2014	Italy	M,W	Caucasian outpatients population-based	4.4	60.7	Measured/Self-reported	Medical records	546	3549	smoking, fasting glucose, LDL-cholesterol, age, gender, systolic blood pressure, body mass index, left ventricular mass index, E/A ratio, left atrial volume index , estimated glomerular filtration rate.	6
Perez, M. V[9]	2013	US	W	Postmenopausal women	9.8	63	Measured/Self-reported	Medical records/ICD coding	8252	81892	Age, hypertension, obesity, diabetes, myocardial infarction and heart failure	8
Suzuki, S[10]	2013	Japan	M,W	Patients attending the cardiovascular institute	7	61	Measured/Self-reported	Medical records	2296	17517	atrial fibrillation and sex	5
Lipworth, L[11]	2012	US	M,W	Population-based	5.7	72	Self-reported	Medical records/ICD coding	1062	8836	race and gender	6
Ninios, I[12]	2010	Greece	M,W	Population-based	2	72.5	Measured/Self-reported	Medical	36	720	Not known	4

								records				
Watanabe, H[13]	2009	Japan	M,W	Community-based	4.5	59.2	Measured/Self-reported	Medical records	265	28449	Age and sex，	6

Abbreviations: M: Men; W: Women; Nos: Newcastle-Ottawa score; AF:atrial fibrillation;ICD：International Classification of Disease

etable 8: Characteristics of Studies Included in the Meta-analysis of AF associated with Triglyceride

Study	Publish Year	country	Sex	Source	Mean Follow-up (y)	Mean age (y)	Exposure confirmation	Outcome Assessment	No. of Cases	No of subjects	NOS
Espnes, H[1]	2021	Northern Norway	M,W	Population-based	17.6	46	Measurement，Self-reported	Medical records	2018	24804	8
Ding, L[2]	2017	China	M,W	population-based	2.6	56.69	Measured	Medical records	134	33186	7
Lim, C. W[3]	2016	Malaysia	M,W	community-based	15	52.6	Measured/Self-reported	Medical records	53	10805	8
Park, H. C[4]	2015	Korea	M,W	Population-based	4	60.2	Measured/Self-reported	Medical records	54	4053	6
Chuang, S. Y[5]	2014	Taiwan	M,W	Population-based	9.16	72.4	Measured	Medical records/ICD coding	90	4053	7
Karppi, J[6]	2013	Finland	M,W	Population-based	2.8	71	Measured/Self-reported	Medical records/ICD coding	105	2340	7
Ravn, L. S[7]	2008	Denmark	M,W	Population-based	26	62	Measured/Self-reported	Medical records/ICD coding	968	9235	6

Abbreviations: M: Men; W: Women; Nos: Newcastle-Ottawa score; AF:atrial fibrillation; ICD：International Classification of Disease

etable 9: Characteristics of Studies Included in the Meta-analysis of AF associated with Total Cholesterol

Study	Publish Year	country	Sex	Source	Mean Follow-up (y)	Mean age (y)	Exposure confirmation	Outcome Assessment	No. of Cases	No of subjects	NOS
Espnes, H[1]	2021	Norway	M,W	Population-based	17.6	46	Measurement，Self-reported	Medical records	2018	24804	8
Garg, P. K[2]	2018	US	W，M	Non-hispanic black and white adults	9.4	63	Measured/Self-reported	Medical records/Self-reported	725	9576	7
Tikhonoff, V[3]	2018	Belgium	W，M	Population-based	14	43.1	Measured/Self-reported	Medical records	143	3956	8
Ding, L[4]	2017	China	M,W	Population-based	2.6	56.69	Measured	Medical records	134	33186	7
Kokubo, Y[5]	2017	Japan	M,W	Population-based	13.8	58.95	Measured/Self-reported	Medical records	311	6898	8
Raman, D[6]	2017	US	M	Older community-dwelling men	8	75.8	Measured/Self-reported	Medical records	269	2350	6
Lim, C. W[7]	2016	Malaysia	M,W	Community-based	15	52.6	Measured/Self-reported	Medical records	53	10805	8
Park, H. C[8]	2015	Korea	M,W	Population-based	4	60.2	Measured/Self-reported	Medical records	54	4053	6
Sciacqua, A[9]	2015	Italy	M,W	Outpatient	4.4	60.7	Measured	Medical records/Self-reported	546	3549	6
Chrispin, J[10]	2014	US	M,W	Population-based	6.9	65.5	Measured/Self-reported	Medical records/ICD coding	214	4942	7
Mora, S[11]	2014	US	W	Healthy female healthcare professionals	16.4	55.65	Measured/Self-reported	Medical records	795	23738	8
Grundvold, I[12]	2012	Norway	M	Population-based	35	50	Measured/Self-reported	Medical records/ICD coding	270	2014	8
Patton, K.	2009	US	M,W	Community-based	10	73.6	Measured/Self-reported	Medical records/ICD coding	1126	5021	7

K[13]											
Ravn, L. S[14]	2008	Denmark	M,W	Population-based	26	62	Measured/Self-reported	Medical records/ICD coding	968	9235	6

Abbreviations: M: Men; W: Women; Nos: Newcastle-Ottawa score; AF:atrial fibrillation; ICD：International Classification of Disease

etable 10: Characteristics of Studies Included in the Meta-analysis of AF associated with High-density lipoprotein

Study	Year	country	Sex	Source	Mean Follow-up (y)	Mean age (y)	Case Assessment	Outcome Assessment	No. of Cases	Total sample size	NOS
Espnes, H[1]	2021	Norway	M,W	Population-based	17.6	46	Measurement，Self-reported	Medical records	2018	24804	8
Ding, L[2]	2017	China	M,W	Population-based	2.6	56.69	Measured	Medical records	134	33186	7
Kokubo, Y[3]	2017	Japan	M,W	Population-based	13.8	58.95	Measured/Self-reported	Medical records	311	6898	8
Lim, C. W[4]	2016	Malaysia	M,W	Community-based	15	52.6	Measured/Self-reported	Medical records	53	10805	8
Park, H. C[5]	2015	Korea	M,W	Population-based	4	60.2	Measured/Self-reported	Medical records	54	4053	6
Sciacqua, A[6]	2015	Italy	M,W	Outpatient	4.4	60.7	Measured	Medical records/Self-reported	546	3549	6
Chrispin, J[7]	2014	US	M,W	Population-based	6.9	65.5	Measured/Self-reported	Medical records/ICD coding	214	4942	7
Chuang, S. Y[8]	2014	Taiwan	M,W	Population-based	9.16	72.4	Measured	Medical records/ICD coding	90	1485	7
Mora, S[9]	2014	US	W	Healthy female healthcare professionals	16.4	55.65	Measured/Self-reported	Medical records	795	23738	8
Agarwal,S. K[10]	2013	US	M,W	Population-based	18.1	55.3	Measured/Self-reported	Medical records/ICD coding	1438	23738	6
Karppi, J[11]	2013	Finland	M,W	Population-based	2.8	71	Measured/Self-reported	Medical records/ICD coding	105	2340	7
Patton, K. K[12]	2009	US	M,W	Community-based	10	73.6	Measured/Self-reported	Medical records/ICD coding	1126	5021	7
Ravn, L. S[13]	2008	Denmark	M,W	Population-based	26	62	Measured/Self-reported	Medical records/ICD coding	968	9235	6

Abbreviations: M: Men; W: Women; Nos: Newcastle-Ottawa score; AF:atrial fibrillation; ICD：International Classification of Disease

etable 11: Characteristics of Studies Included in the Meta-analysis of AF associated with Low-density lipoprotein

Study	Year	country	Sex	Source	Mean Follow-up (y)	Mean age (y)	Case Assessment	Outcome Assessment	No. of Cases	Total sample size	NOS
Ding, L[1]	2017	China	M,W	Population-based	2.6	56.69	Measured	Medical records	134	33186	7
Perkiömäki, J. S[2]	2017	Finland	M,W	Population-based	16.4	51.95	Measured	Based on ICD code	91	903	7
Lim, C. W[3]	2016	Malaysia	M,W	Community-based	15	52.6	Measured/Self-reported	Medical records	53	10805	8
Sciacqua, A[4]	2015	Italy	M,W	Outpatient	4.4	60.7	Measured	Medical records/Self-reported	546	3549	6
Mora, S[5]	2014	US	W	Healthy female healthcare professionals	16.4	55.65	Measured/Self-reported	Medical records	795	23738	8
Agarwal, S. K[6]	2013	US	M,W	Population-based	18.1	55.3	Measured/Self-reported	Medical records/ICD coding	1438	12071	6
Karppi, J[7]	2013	Finland	M,W	Population-based	2.8	71	Measured/Self-reported	Medical records/ICD coding	105	2340	7

Abbreviations: M: Men; W: Women; Nos: Newcastle-Ottawa score; AF:atrial fibrillation; ICD：International Classification of Disease

etable 12: Sensitivity and heterogeneity analysis of pooled RR of AF for obesity and increased BMI

	Obesity				BMI (Per 5 unit increasement)			
	**n studies**	**RR (95% CI)**	**I^2**	**P-Value**	**n studies**	**RR (95% CI)**	**I^2**	**P-Value**
**Statistical model**								
Random effects	37	1.39(1.30, 1.49)	85.9%	0.00	31	1.27(1.22, 1.32)	90.5%	0.00
Fixed effects	37	1.32(1.30, 1.35)			31	1.22(1.20, 1.22)		
**Analysis of all studies with**								
Population/community-based	24	1.51(1.45, 1.57)	4.6%	0.40	25	1.29(1.23，1.34)	83.1%	0.00
Measurement	33	1.40(1.31, 1.51)	84.9%	0.00	30	1.27(1.21, 1.32)	90.6%	0.00
High-quality (NOS ≥ 6)	34	1.41(1.31, 1.51)	84.2%	0.00	29	1.27(1.22, 1.33)	91.1%	0.00
Mean follow-up ≥ 10 y	19	1.45(1.31, 1.60)	89.2%	0.00	21	1.30(1.24, 1.36)	87.4%	0.00
Adjusted risk estimate [a]	27	1.41(1.30, 1.53)	84.9%	0.00	26	1.25(1.20, 1.31)	91.2%	0.00
Large cohort [b]	18	1.41(1.29, 1.54)	92.6%	0.00	13	1.29(1.22, 1.37)	95.2%	0.00
**Analysis of all studies except**								
Two largest studies [c]	35	1.37(1.29, 1.46)	80.0%	0.00	29	1.27(1.22, 1.32)	89.5%	0.00
One outlier study [d]	36	1.38(1.29, 1.48)	86.1%	0.00	30	1.25(1.20, 1.30)	88.7%	0.00

Abbreviations: RR: Relative Risk; AF: atrial fibrillation; BMI: Body Mass Index; Nos: Newcastle-Ottawa score;

[a] Studies reporting estimates that adjusted for at least three confounding factors.

[b] Large prospective cohort studies with sample size over 15,000.

[c] Studies with population over 1 million.

[d] Studies with largest RR.

etable 13: Sensitivity and heterogeneity analysis of pooled WMD of AF for BMI

	BMI			
	**n studies**	**WMD (95% CI)**	**I^2**	**P-Value**
**Statistical model**				
Random effects	28	0.74(0.52, 0.95)	90.2%	0.00
Fixed effects	28	0.61(0.56, 0.66)		
**Analysis of all studies with**				
Population/community-based	23	0.78(0.48, 1.08)	89.2%	0.00
Measurement	27	0.72(0.50, 0.94)	90.3%	0.00
High-quality (NOS ≥ 6)	27	0.75(0.53, 0.96)	90.5%	0.00
Mean follow-up ≥ 10 y	15	0.92(0.63, 1.21)	92.5%	0.00
Adjusted risk estimate [a]	18	0.82(0.54, 1.09)	89.0%	0.00
Large cohort [b]	5	0.47(0.15, 0.79)	94.6%	0.00

Abbreviations: RR: Relative Risk; AF: atrial fibrillation; BMI: Body Mass Index; Nos: Newcastle-Ottawa score;

[a] Studies reporting estimates that adjusted for at least three confounding factors.

[b] Large prospective cohort studies with sample size over 15,000.

etable 14: Sensitivity and heterogeneity analysis of pooled RR of AF for Smokers

	Former Versus Never Smokers				Current Versus Never Smoker			
	**n studies**	**RR (95% CI)**	**I^2**	**P-Value**	**n studies**	**RR (95% CI)**	**I^2**	**P-Value**
**Statistical model**								
Random effects	16	1.19(1.10, 1.28)	43.3%	0.03	18	1.23(1.09, 1.38)	75.0%	0.00
Fixed effects	16	1.18(1.14, 1.22)			18	1.26(1.20, 1.32)		
**Analysis of all studies with**								
Population-based	13	1.20(1.07, 1.34)	53.2%	0.01	16	1.19(1.04, 1.37)	76.7%	0.00
High-quality (NOS ≥ 6)	15	1.18(1.10, 1.27)	45.4%	0.02	17	1.24(1.10, 1.39)	75.9%	0.00
Mean follow-up ≥ 10 y	8	1.17(1.08, 1.26)	1.9%	0.42	9	1.26(1.03, 1.54)	82.1%	0.00
Adjusted risk estimate [a]	13	1.19(1.10, 1.30)	51.6%	0.02	15	1.28(1.14, 1.44)	74.2%	0.00
Large cohort [b]	4	1.14(0.97, 1.35)	74.0%	0.01	5	1.38(1.11, 1.72)	89.8%	0.00
**Analysis of all studies except**								
Two largest studies [c]	16	1.19(1.10, 1.28)	43.3%	0.03	18	1.23(1.09, 1.38)	75.0%	0.00
One outlier study [d]	15	1.17(1.09, 1.26)	36.4%	0.08	17	1.18(1.07, 1.30)	59.5%	0.00

Abbreviations: RR: Relative Risk; AF: atrial fibrillation;Nos: Newcastle-Ottawa score;

[a] Studies reporting estimates that adjusted for at least three confounding factors.

[b] Large prospective cohort studies with sample size over 15,000.

[c] Studies with population over 1 million.

[d] Studies with largest RR.

etable 15: Sensitivity and heterogeneity analysis of pooled RR of AF for Diabetes and Hypertension

	Diabetes				Hypertension			
	**n studies**	**RR (95% CI)**	**I^2**	**P-Value**	**n studies**	**RR (95% CI)**	**I^2**	**P-Value**
**Statistical model**								
Random effects	48	1.31(1.23, 1.39)	87.8%	0.00	44	1.68(1.51, 1.87)	99.3%	0.00
Fixed effects	48	1.11(1.10, 1.12)			44	1.51(1.50, 1.52)		
**Analysis of all studies with**								
Population/community-based	35	1.29(1.20, 1.39)	87.3%	0.00	33	1.69(1.58, 1.80)	96.6%	0.00
Measurement	39	1.37(1.26, 1.49)	80.4%	0.00	36	1.69(1.39, 2.07)	99.0%	0.00
High-quality (NOS ≥ 6)	43	1.32(1.23, 1.41)	88.7%	0.00	39	1.68(1.43, 1.97)	99.4%	0.00
Mean follow-up ≥ 10 y	22	1.43(1.24, 1.45)	90.5%	0.00	20	1.73(1.31, 2.28)	99.7%	0.00
Adjusted risk estimate [a]	35	1.34(1.15, 1.33)	90.4%	0.00	31	1.70(1.40, 2.05)	99.5%	0.00
Large cohort [b]	20	1.30(1.20, 1.42)	93.6%	0.00	19	1.83(1.56, 2.13)	99.7%	0.00
**Analysis of all studies except**								
Two largest studies [c]	46	1.32(1.24, 1.41)	81.1%	0.00	42	1.69(1.42, 2.00)	98.9%	0.00
One outlier study [d]	47	1.27(1.20, 1.34)	82.4%	0.00	43	1.68(1.51, 1.87)	99.3%	0.00

Abbreviations: RR: Relative Risk; AF: atrial fibrillation; Nos: Newcastle-Ottawa score;

[a] Studies reporting estimates that adjusted for at least three confounding factors.

[b] Large prospective cohort studies with sample size over 15,000.

[c] Studies with population over 1 million.

[d] Studies with largest RR.

etable 16: Sensitivity and heterogeneity analysis of pooled RR of AF for Dyslipidemia

	Dyslipidemia			
	n studies	RR (95% CI)	I^2	P-Value
**Statistical model**				
Random effects	13	1.12(0.95, 1.32)	92.9%	0.00
Fixed effects	13	0.91(0.88, 0.94)		
**Analysis of all studies with**				
Population-based	9	1.23(0.91, 1.66)	94.8%	0.00
High-quality (NOS ≥ 6)	11	1.12(0.95, 1.33)	94.0%	0.00
Mean follow-up ≥ 5 y	8	1.01(0.86, 1.19)	93.2%	0.00
Adjusted risk estimate [a]	9	1.24(0.99, 1.55)	94.8%	0.00
Large cohort [b]	6	0.89(0.77, 1.03)	87.8%	0.00
**Analysis of all studies except**				
Two largest studies [c]	13	1.12(0.95, 1.32)	92.9%	0.00
One outlier study [d]	12	1.02(0.88, 1.17)	90.1%	0.00

Abbreviations: RR: Relative Risk; AF: atrial fibrillation; Nos: Newcastle-Ottawa score;

[a] Studies reporting estimates that adjusted for at least three confounding factors.

[b] Large prospective cohort studies with sample size over 15,000.

[c] Studies with population over 1 million.

[d] Studies with largest RR.

etable 17: Sensitivity and heterogeneity analysis of pooled WMD of AF for Triglyceride and Total Cholesterol

	Triglyceride				Total Cholesterol			
	**n studies**	**WMD (95% CI)**	**I^2**	**P-Value**	**n studies**	**WMD (95% CI)**	**I^2**	**P-Value**
**Statistical model**								
Random effects	7	0.00(-0.08, 0.09)	67.6%	0.00	14	-0.02(-0.10, 0.06)	89.4%	0.00
Fixed effects	7	0.06(0.02, 0.10)			14	-0.01(-0.04, 0.01)		
**Analysis of all studies with**								
Population-based	7	0.00(-0.08, 0.09)	67.6%	0.00	11	-0.04(-0.13, 0.05)	86.9%	0.00
High-quality (NOS ≥ 6)	7	0.00(-0.08, 0.09)	67.6%	0.00	14	-0.02(-0.10, 0.06)	89.4%	0.00
Mean follow-up ≥ 10 y	3	0.11(0.06, 0.15)	0.00%	0.44	8	0.03(-0.08, 0.14)	90.9%	0.00
Adjusted risk estimate [a]	3	0.00(-0.17, 0.16)	82.5%	0.00	10	0.00(-0.08, 0.09)	87.8%	0.00
Large cohort [b]	2	0.06(-0.09, 0.20)	67.6%	0.08	3	0.03(-0.08, 0.16)	85.4%	0.00

Abbreviations: RR: Relative Risk; AF: atrial fibrillation; Nos: Newcastle-Ottawa score;

[a] Studies reporting estimates that adjusted for at least three confounding factors.

[b] Large prospective cohort studies with sample size over 15,000.

etable 18: Sensitivity and heterogeneity analysis of pooled WMD of AF for High-density lipoprotein and Low-density lipoprotein

	High-density lipoprotein				Low-density lipoprotein			
	**n studies**	**WMD (95% CI)**	**I^2**	**P-Value**	**n studies**	**WMD (95% CI)**	**I^2**	**P-Value**
**Statistical model**								
Random effects	13	-0.05(-0.07, -0.02)	81.6%	0.00	7	0.02(-0.05, 0.09)	61.8%	0.02
Fixed effects	13	-0.05(-0.06, -0.04)			7	0.05(0.01, 0.08)		
**Analysis of all studies with**								
Population-based	11	-0.06(-0.08, -0.03)	72.7%	0.00	5	-0.04(-0.15, 0.08)	64.9%	0.02
High-quality (NOS ≥ 6)	13	-0.05(-0.07, -0.02)	81.6%	0.00	7	0.02(-0.05, 0.09)	61.8%	0.02
Mean follow-up ≥ 10 y	7	-0.05(-0.09, -0.02)	87.7%	0.00	4	0.02(-0.07, 0.12)	70.5%	0.02
Adjusted risk estimate [a]	7	-0.03(-0.05, 0.00)	67.7%	0.00	4	0.07(0.02, 0.12)	10.7%	0.34
Large cohort [b]	4	-0.04(-0.09, 0.00)	67.7%	0.00	2	0.03(-0.08, 0.15)	64.5%	0.09

Abbreviations: RR: Relative Risk; AF: atrial fibrillation; Nos: Newcastle-Ottawa score;

[a] Studies reporting estimates that adjusted for at least three confounding factors.

[b] Large prospective cohort studies with sample size over 15,000.

etable 19: The quality assessment of included cohort studies assessing the association between obesity and AF using the Newcastle-Ottawa scale

	**Selection**				**Comparability**		**Outcome**			
Author	Representativeness of Exposed Cohort	Selection of Non-Exposed Cohort	Ascertainment Of Exposure	Demonstration That Outcome of Interest Was Not Present at Start of Study	Adjust for age	Adjust for 3 or more important confounders (smoking, alcohol consumption, physical activity, diet)	Assessment of outcome	Follow-up Length≥ 10years	Loss to follow-up rate≤ 20%	Total Quality Score
Denas, G 2020	1	1	1	1	1	1	1	0	0	7

Singleton, M. J 2021	1	1	1	1	1	1	1	1	0	8
Tsai, S. Y 2021	1	1	1	1	1	1	1	1	0	8
Matsuo.K 2012	1	1	0	1	0	0	1	1	1	6
Johansson, C 2020	1	1	1	1	1	1	1	1	0	8
Kamil-Rosenberg, S 2020	0	1	1	1	0	0	1	1	0	5
Younis, A 2020	0	1	1	1	1	1	1	0	0	6
Ball, J 2018	1	1	1	1	1	1	1	1	1	9
Crump, C 2018	1	1	1	1	1	1	1	1	1	9
Di Benedetto, L 2018	1	1	1	1	1	1	1	1	1	9
Foy, A. J 2018	1	1	1	1	1	1	1	0	0	7
Garnvik, L. E 20018	1	1	1	1	1	1	1	0	1	8
Persson, C. E 2017	1	1	1	1	1	1	1	1	1	9
Kokubo, Y 2017	1	1	1	1	1	1	1	0	1	8
O'Neal, W. T 2017	1	1	1	1	1	1	1	0	0	7
Trevisan, C 2917	1	1	1	1	1	1	1	0	0	7
Diouf, I 2016	1	1	1	1	1	1	1	0	0	7
Mazzone, C 2015	0	1	1	1	0	0	1	0	0	4
Nyström, P. K 2015	1	1	1	1	1	1	1	1	1	9
Sciacqua, A 2015	0	1	1	1	1	1	1	0	0	6
Vermond, R. A 2015	1	1	1	1	1	1	1	0	1	8
Vermond, R. A 2014	0	1	1	1	1	1	1	1	1	8
Chuang, S. Y 2014	1	1	1	1	1	1	1	0	0	7
Schmidt, M 2014	1	1	1	1	0	0	1	1	1	7
Karasoy, D 2013	1	1	1	1	1	1	1	0	1	8

Suzuki, S 2013	1	1	1	1	0	0	1	0	0	5
Grundvold, I 2012	1	1	1	1	1	1	1	1	0	8
Lipworth, L 2012	1	1	1	1	1	0	1	0	0	6
Chamberlain, A. M 2011	1	1	1	1	1	0	1	1	1	8
Smith, J. G 2010	1	1	1	1	1	0	1	1	1	8
Tedrow, U. B 2010	1	1	1	1	1	1	1	1	1	9
Nichols, G. A 2009	1	1	1	1	1	0	1	0	1	7
Watanabe, H 2008	1	1	1	1	1	0	1	0	0	6
Umetani, K 2007	0	1	1	1	0	0	1	0	0	4
Frost, L 2005	1	1	1	1	1	1	1	0	1	8
Wang, T. J 2004	1	1	1	1	1	1	1	1	1	9
Wilhelmsen, L 2001	1	1	1	1	1	0	1	1	0	7
Krahn, A. D 1995	0	1	1	1	1	1	1	1	0	7

etable 20: The quality assessment of included cohort studies assessing the association between Per 5kg/m^2 Increase in BMI and AF using the Newcastle-Ottawa scale

	Selection				Comparability		Outcome			
Author	Representativeness of Exposed Cohort	Selection of Non-Exposed Cohort	Ascertainment Of Exposure	Demonstration That Outcome of Interest Was Not Present at Start of Study	Adjust for age	Adjust for 3 or more important confounders (smoking, alcohol consumption, physical activity, diet)	Assessment of outcome	Follow-up Length≥ 10years	Loss to follow-up rate≤ 20%	Total Quality Score
Matsumoto, K 2021	1	1	1	1	1	1	1	0	1	8
Singleton, M. J 2021	1	1	1	1	1	1	1	1	0	8
Zia, I 2021	1	1	1	1	1	1	1	1	1	9
Johansson, C 2020	1	1	1	1	1	1	1	1	0	8
Schrage, B 2020	1	1	1	1	0	0	1	1	1	7
Berkovitch, A 2019	0	1	1	1	0	1	1	0	0	5
Aronson, D 2018	1	1	1	1	1	1	1	1	1	9
Crump, C 2018	1	1	1	1	1	1	1	1	1	9
Persson, C. E 2017	1	1	1	1	1	1	1	1	1	9
Ding, L 2017	1	1	1	1	1	1	1	0	0	7
Magnussen, C 2017	1	1	1	1	1	1	1	1	0	8
Trevisan, C 2917	1	1	1	1	1	1	1	0	0	7
Karas, M. G 2016	1	1	1	1	1	1	1	1	0	8
Dewland, T. A 2015	1	1	1	1	1	1	1	1	1	9
Käräjämäki,A.J 2015	1	1	1	1	1	1	1	1	1	9
Kokubo, Y 2015	1	1	1	1	1	1	1	1	1	9
Nyström, P. K 2015	1	1	1	1	1	1	1	1	1	9
Park, H. C 2015	1	1	1	1	0	0	1	0	1	6

Sciacqua, A 2015	0	1	1	1	1	1	1	0	0	6
Vermond, R. A 2015	1	1	1	1	1	1	1	0	1	8
Knuiman, M 2014	1	1	1	1	1	1	1	1	1	9
Mandalenakis,Z 2014	1	1	1	1	1	1	1	1	1	9
Schmidt, M 2014	1	1	1	1	0	0	1	1	1	7
Grundvold, I. 2013	0	1	1	1	1	1	1	1	0	7
Nyrnes, A 2013	1	1	1	1	1	1	1	1	1	9
Perez, M. V 2013	1	1	1	1	1	1	1	0	1	8
Casaclang-Verzosa, G 2010	0	1	1	1	1	0	1	0	0	5
Tedrow, U. B 2010	1	1	1	1	1	1	1	1	1	9
Frost, L 2005	1	1	1	1	1	1	1	0	1	8
Wang, T. J 2004	1	1	1	1	1	1	1	1	1	9
Wilhelmsen, L 2001	1	1	1	1	1	0	1	1	0	7

etable 21: The quality assessment of included cohort studies assessing the association between BMI and AF using the Newcastle-Ottawa scale

	**Selection**				**Comparability**		**Outcome**			
Author	Representativeness of Exposed Cohort	Selection of Non-Exposed Cohort	Ascertainment Of Exposure	Demonstration That Outcome of Interest Was Not Present at Start of Study	Adjust for age	Adjust for 3 or more important confounders (smoking, alcohol consumption, physical activity, diet)	Assessment of outcome	Follow-up Length≥ 10years	Loss to follow-up rate≤ 20%	Total Quality Score
Denas, G 2020	1	1	1	1	1	1	1	0	0	7
Espnes, H 2021	1	1	1	0	1	1	1	1	1	8

Bundy, J. D 2020	1	1	1	1	1	1	1	1	0	8
Persson, A. P 2019	1	1	1	1	1	1	1	1	0	8
Andersen, K 2018	0	1	1	1	0	1	1	1	1	7
Garg, P. K 2018	1	1	1	1	1	1	1	0	0	7
Tikhonoff, V 2018	1	1	1	1	1	1	1	1	0	8
Ding, L 2017	1	1	1	1	1	1	1	0	0	7
Kokubo, Y 2017	1	1	1	1	1	1	1	0	1	8
Perkiömäki,J.S 2017	1	1	1	1	1	1	1	1	1	9
Raman, D 2017	0	1	1	1	1	1	1	0	0	6
Trevisan, C 2917	1	1	1	1	1	1	1	0	0	7
Ermakov, S 2016	0	1	1	1	1	1	1	1	1	8
Lim, C. W 2016	1	1	1	1	1	0	1	1	1	8
Nyström, P.K 2015	1	1	1	1	1	1	1	1	1	9
Park, H. C 2015	1	1	1	1	0	0	1	0	1	6
Sciacqua, A 2015	0	1	1	1	1	1	1	0	0	6
Vermond,R.A 2015	1	1	1	1	1	1	1	0	1	8
Chuang, S. Y 2014	1	1	1	1	1	1	1	0	0	7
McManus, D. D 2014	1	1	1	1	0	0	1	0	1	6
Agarwal, S.K 2013	1	1	1	1	0	0	1	1	0	6
Karppi, J 2013	1	1	1	1	0	0	1	1	1	7
Suzuki, S 2013	1	1	1	1	0	0	1	0	0	5
Grundvold, I 2012	1	1	1	1	1	1	1	1	0	8
Ninios, I 2010	0	1	1	1	0	0	1	0	0	4
Patton, K. K 2009	1	1	1	1	0	0	1	1	1	7

Ravn, L. S 2008	1	1	1	1	0	0	1	1	0	6
Watanabe, H 2006	1	1	1	1	0	0	1	1	0	6

etable 22: The quality assessment of included cohort studies assessing the association between smoking and AF using the Newcastle-Ottawa scale

	**Selection**				**Comparability**		**Outcome**			
Author	Representativeness of Exposed Cohort	Selection of Non-Exposed Cohort	Ascertainment Of Exposure	Demonstration That Outcome of Interest Was Not Present at Start of Study	Adjust for age	Adjust for 3 or more important confounders (smoking, alcohol consumption, physical activity, diet)	Assessment of outcome	Follow-up Length ≥ 10 years	Loss to follow-up rate ≤ 20%	Total Quality Score
Banks, E 2019	1	1	1	1	1	1	1	0	0	7
Zuo, H 2018	1	1	1	1	1	1	1	1	1	9
Kokubo,Y 2017	1	1	1	1	1	1	1	0	1	8
Diouf, I 2016	1	1	1	1	1	1	1	0	0	7
Lim, C. W 2016	1	1	1	1	1	0	1	1	1	8
Dewland, T. A 2015	1	1	1	1	1	1	1	1	1	9
Suzuki, S 2015	1	1	1	1	1	1	1	0	1	8
Tiwari, S 2015	1	1	1	1	0	0	1	1	0	6
Adamsson, E. S 2014	1	1	1	1	1	1	1	1	0	8
Chrispin,J 2014	1	1	1	1	1	1	1	0	0	7
Chuang,S.Y 2014	1	1	1	1	1	1	1	0	0	7
Knuiman,M 2014	1	1	1	1	1	1	1	1	1	9
Perez,M.V 2013	1	1	1	1	1	1	1	0	1	8
Chamberlain, A. M 2011	1	1	1	1	1	1	1	1	1	9
Ninios, I 2010	0	1	1	1	0	0	1	0	0	4

Rosengren,A 2009	1	1	1	1	1	0	1	1	1	8
Heeringa,J 2008	1	1	1	1	1	1	1	0	0	7
Frost, L 2005	1	1	1	1	1	1	1	0	1	8

etable 23: The quality assessment of included cohort studies assessing the association between diabetes and AF using the Newcastle-Ottawa scale

	Selection				Comparability		Outcome			
Author	Representativeness of Exposed Cohort	Selection of Non-Exposed Cohort	Ascertainment Of Exposure	Demonstration That Outcome of Interest Was Not Present at Start of Study	Adjust for age	Adjust for 3 or more important confounders (smoking, alcohol consumption, physical activity, diet)	Assessment of outcome	Follow-up Length ≥ 10 years	Loss to follow-up rate ≤ 20%	Total Quality Score
Chao, T. F 2021	1	1	1	1	1	1	1	1	0	8
Denas, G 2020	1	1	1	1	1	1	1	0	0	7
Espnes, H 2021	1	1	1	0	1	1	1	1	1	8
Himmelreich,J 2021	1	1	1	1	1	0	1	0	0	6
Viktor Lind 2021	1	1	1	1	1	1	1	1	0	8
Matsumoto,K 2020	1	1	1	1	1	1	1	0	1	8
Schmidt, C 2021	0	1	1	1	1	0	1	0	0	5
Schrage, B 2020	1	1	1	1	0	0	1	1	1	7
Staerk, L 2020	1	1	1	1	1	1	1	1	1	9
Younis, A 2020	0	1	1	1	1	1	1	0	0	6
Li, Y. G 2019	1	1	1	1	1	1	1	0	0	7
Austin, T. R 2018	1	1	1	1	1	1	1	0	1	8
Crump, C 2018	1	1	1	1	1	1	1	1	1	9
Foy, A. J 2018	1	1	1	1	0	0	0	0	0	4
Tikhonoff, V 2018	1	1	1	1	1	1	1	1	0	8
Ding, L 2017	1	1	1	1	1	1	1	0	0	7
Gillott, R. G	1	1	1	1	1	1	1	0	0	7
Kokubo, Y 2017	1	1	1	1	1	1	1	0	1	8

Magnussen,C 2017	1	1	1	1	1	1	1	1	0	8
O'Neal, W. T 2017	1	1	1	1	1	1	1	0	0	7
Raman, D 2017	0	1	1	1	1	1	1	0	0	6
Trevisan, C 2917	1	1	1	1	1	0	1	0	0	6
Diouf, I 2016	1	1	1	1	1	1	1	0	0	7
Lim, C. W 2016	1	1	1	1	1	0	1	1	1	8
Dewland, T.A 2015	1	1	1	1	1	1	1	1	1	9
Käräjämäki,A.J 2015	1	1	1	1	1	1	1	1	1	9
Mazzone, C 2015	0	1	1	1	0	0	1	0	0	4
Sciacqua, A 2015	0	1	1	1	1	1	1	0	0	6
Vermond, R. A 2015	1	1	1	1	1	1	1	0	1	8
Chrispin, J 2014	1	1	1	1	1	1	1	0	0	7
Frost, L 2014	1	1	1	1	1	1	1	1	1	9
Knuiman, M 2014	1	1	1	1	1	1	1	1	1	9
Mora, S 2014	0	1	1	1	1	1	1	1	1	8
Karppi, J 2013	1	1	1	1	0	0	1	1	1	7
Perez, M. V 2013	1	1	1	1	1	1	1	0	1	8
Suzuki, S 2013	1	1	1	1	0	0	1	0	0	5
Lipworth, L 2012	1	1	1	1	1	0	1	0	0	6
Chamberlain, A. M 2011	1	1	1	1	1	0	1	1	1	8
Casaclang-Verzosa, G 2010	0	1	1	1	1	0	1	0	0	5
Ninios, I 2010	0	1	1	1	0	0	1	0	0	4

Smith, J. G 2010	1	1	1	1	1	0	1	1	1	8
Nichols, G. A 2009	1	1	1	1	1	0	1	0	1	7
Patton, K. K 2009	1	1	1	1	0	0	1	1	1	7
Rosengren, A 2009	1	1	1	1	1	0	1	1	1	8
Umetani, K 2007	0	1	1	1	0	0	1	0	0	4
Watanabe, H 2006	1	1	1	1	0	0	1	1	0	6
Friberg, J 2003	1	1	1	1	1	1	1	0	0	7
Krahn, A. D 1995	0	1	1	1	1	0	1	1	0	6

etable 24: The quality assessment of included cohort studies assessing the association between hypertension and AF using the Newcastle-Ottawa scale

	Selection				Comparability		Outcome			
Author	Representativeness of Exposed Cohort	Selection of Non-Exposed Cohort	Ascertainment Of Exposure	Demonstration That Outcome of Interest Was Not Present at Start of Study	Adjust for age	Adjust for 3 or more important confounders (smoking, alcohol consumption, physical activity, diet)	Assessment of outcome	Follow-up Length≥ 10years	Loss to follow-up rate≤ 20%	Total Quality Score
Chao, T. F 2021	1	1	1	1	1	1	1	1	0	8
Denas, G 2020	1	1	1	1	1	1	1	0	0	7
Espnes, H 2021	1	1	1	0	1	1	1	1	1	8
Himmelreich,J 2021	1	1	1	1	1	0	1	0	0	6
Matsumoto,K 2020	1	1	1	1	1	1	1	0	1	8
Lip, G 2020	1	1	1	1	0	0	1	0	0	5
Niklas, A 2020	1	1	1	1	0	0	1	0	0	5
Younis, A 2020	0	1	1	1	1	1	1	0	0	6
Li, Y. G 2019	1	1	1	1	1	1	1	0	0	7
Rattani, A 2019	1	1	1	1	1	1	1	1	1	9
Aronson, D 2018	1	1	1	1	1	1	1	1	1	9
Crump, C 2018	1	1	1	1	1	1	1	1	1	9
Foy, A. J 2018	1	1	1	1	0	0	0	0	0	4
Ding, L 2017	1	1	1	1	1	1	1	0	0	7
Gillott, R. G	1	1	1	1	1	1	1	0	0	7
O'Neal, W. T 2017	1	1	1	1	1	1	1	0	0	7
Perkiömäki,J.S 2017	1	1	1	1	1	1	1	1	1	9
Raman, D 2017	0	1	1	1	1	1	1	0	0	6

Trevisan, C 2917	1	1	1	1	1	0	1	0	0	6
Diouf, I 2016	1	1	1	1	1	1	1	0	0	7
Lim, C. W 2016	1	1	1	1	1	0	1	1	1	8
Dewland, T.A 2015	1	1	1	1	1	1	1	1	1	9
Khan, H 2015	1	1	1	1	1	1	1	1	0	8
Kokubo, Y 2015	1	1	1	1	1	1	1	1	1	9
Mazzone, C 2015	0	1	1	1	0	0	1	0	0	4
Nyström,P. K 2015	1	1	1	1	1	1	1	1	1	9
O'Neal, W. T 2015	1	1	1	1	1	1	1	0	0	7
Sciacqua, A 2015	0	1	1	1	1	1	1	0	0	6
Torén, K 2015	1	1	1	1	1	1	1	0	1	8
Vermond, R. A 2015	1	1	1	1	1	1	1	0	1	8
Frost, L 2014	1	1	1	1	1	1	1	1	1	9
Mora, S 2014	0	1	1	1	1	1	1	1	1	8
Perez, M. V 2013	1	1	1	1	1	1	1	0	1	8
Suzuki, S 2013	1	1	1	1	0	0	1	0	0	5
Lipworth, L 2012	1	1	1	1	1	0	1	0	0	6
Ninios, I 2010	0	1	1	1	0	0	1	0	0	4
Smith, J. G 2010	1	1	1	1	1	0	1	1	1	8
Nichols, G. A 2009	1	1	1	1	1	0	1	0	1	7
Schnabel, R. B 2009	1	1	1	1	1	0	1	1	0	7
Ravn, L. S 2008	1	1	1	1	0	0	1	1	0	6
Umetani, K 2007	0	1	1	1	0	0	1	0	0	4
Watanabe, H 2006	1	1	1	1	0	0	1	1	0	6
Krahn, A. D 1995	0	1	1	1	1	1	1	1	0	7

Lake, F. R 1989	1	1	1	1	1	1	1	0	0	7

etable 25: The quality assessment of included cohort studies assessing the association between Dyslipidemia and AF using the Newcastle-Ottawa scale

	**Selection**				**Comparability**		**Outcome**			
Author	Representativeness of Exposed Cohort	Selection of Non-Exposed Cohort	Ascertainment Of Exposure	Demonstration That Outcome of Interest Was Not Present at Start of Study	Adjust for age	Adjust for 3 or more important confounders (smoking, alcohol consumption, physical activity, diet)	Assessment of outcome	Follow-up Length≥ 10years	Loss to follow-up rate≤ 20%	Total Quality Score
Himmelreich,J 2021	1	1	1	1	1	0	1	0	0	6
Matsumoto, K 2020	1	1	1	1	1	1	1	0	1	8
Schmidt, C 2021	0	1	1	1	1	0	1	0	0	5
Younis, A 2020	0	1	1	1	1	1	1	0	0	6
Li, Y. G 2019	1	1	1	1	1	1	1	0	0	7
O'Neal, W. T 2017	1	1	1	1	1	1	1	0	0	7
Vermond,R.A 2015	1	1	1	1	1	1	1	0	1	8
Sciacqua, A 2014	0	1	1	1	1	1	1	0	0	6
Perez, M. V 2013	1	1	1	1	1	1	1	0	1	8
Suzuki, S 2013	1	1	1	1	0	0	1	0	0	5
Lipworth, L 2012	1	1	1	1	1	0	1	0	0	6
Ninios, I 2010	0	1	1	1	0	0	1	0	0	4
Watanabe, H 2008	1	1	1	1	1	0	1	0	0	6

etable 26: The quality assessment of included cohort studies assessing the association between triglyceride and AF using the Newcastle-Ottawa scale

	Selection				Comparability		Outcome			
Author	Representativeness of Exposed Cohort	Selection of Non-Exposed Cohort	Ascertainment Of Exposure	Demonstration That Outcome of Interest Was Not Present at Start of Study	Adjust for age	Adjust for 3 or more important confounders (smoking, alcohol consumption, physical activity, diet)	Assessment of outcome	Follow-up Length≥ 10years	Loss to follow-up rate≤ 20%	Total Quality Score
Espnes, H 2021	1	1	1	0	1	1	1	1	1	8
Ding, L 2017	1	1	1	1	1	1	1	0	0	7
Lim, C. W 2016	1	1	1	1	1	0	1	1	1	8
Park, H. C 2015	1	1	1	1	0	0	1	0	1	6
Chuang, S. Y 2014	1	1	1	1	1	1	1	0	0	7
Karppi, J 2013	1	1	1	1	0	0	1	1	1	7
Ravn, L. S 2008	1	1	1	1	0	0	1	1	0	6

etable 27: The quality assessment of included cohort studies assessing the association between total cholesterol and AF using the Newcastle-Ottawa scale

	Selection				Comparability		Outcome			
Author	Representativeness of Exposed Cohort	Selection of Non-Exposed Cohort	Ascertainment Of Exposure	Demonstration That Outcome of Interest Was Not Present at Start of Study	Adjust for age	Adjust for 3 or more important confounders (smoking, alcohol consumption, physical activity, diet)	Assessment of outcome	Follow-up Length≥ 10years	Loss to follow-up rate≤ 20%	Total Quality Score
Espnes, H 2021	1	1	1	0	1	1	1	1	1	8
Garg, P. K 2018	1	1	1	1	1	1	1	0	0	7
Tikhonoff, V 2018	1	1	1	1	1	1	1	1	0	8
Ding, L 2017	1	1	1	1	1	1	1	0	0	7
Kokubo, Y 2017	1	1	1	1	1	1	1	0	1	8
Raman, D 2017	0	1	1	1	1	1	1	0	0	6
Lim, C. W 2016	1	1	1	1	1	0	1	1	1	8
Park, H. C 2015	1	1	1	1	0	0	1	0	1	6
Sciacqua, A 2015	0	1	1	1	1	1	1	0	0	6
Chrispin, J 2014	1	1	1	1	1	1	1	0	0	7
Mora, S 2014	0	1	1	1	1	1	1	1	1	8
Grundvold, I 2012	1	1	1	1	1	1	1	1	0	8
Patton, K. K 2009	1	1	1	1	0	0	1	1	1	7
NO.434 Ravn, L. S 2008	1	1	1	1	0	0	1	1	0	6

etable 28: The quality assessment of included cohort studies assessing the association between Low-density lipoprotein and AF using the Newcastle-Ottawa scale

	Selection				Comparability		Outcome			
Author	Representativeness of Exposed Cohort	Selection of Non-Exposed Cohort	Ascertainment Of Exposure	Demonstration That Outcome of Interest Was Not Present at Start of Study	Adjust for age	Adjust for 3 or more important confounders (smoking, alcohol consumption, physical activity, diet)	Assessment of outcome	Follow-up Length≥ 10years	Loss to follow-up rate≤ 20%	Total Quality Score
Ding, L 2017	1	1	1	1	1	1	1	0	0	7
Perkiömäki,J.S 2017	1	1	1	1	0	0	1	1	1	7
Lim, C. W 2016	1	1	1	1	1	0	1	1	1	8
Sciacqua, A 2015	0	1	1	1	1	1	1	0	0	6
Mora, S 2014	0	1	1	1	1	1	1	1	1	8
Agarwal, S. K 2013	1	1	1	1	0	0	1	1	0	6
Karppi, J 2013	1	1	1	1	0	0	1	1	1	7

etable 29: The quality assessment of included cohort studies assessing the association between High-density lipoprotein and AF using the Newcastle-Ottawa scale

	Selection				Comparability		Outcome			
Author	Representativeness of Exposed Cohort	Selection of Non-Exposed Cohort	Ascertainment Of Exposure	Demonstration That Outcome of Interest Was Not Present at Start of Study	Adjust for age	Adjust for 3 or more important confounders (smoking, alcohol consumption, physical activity, diet)	Assessment of outcome	Follow-up Length≥ 10years	Loss to follow-up rate≤ 20%	Total Quality Score
Espnes, H 2021	1	1	1	0	1	1	1	1	1	8
Ding, L 2017	1	1	1	1	1	1	1	0	0	7
Kokubo, Y 2017	1	1	1	1	1	1	1	0	1	8
Lim, C. W 2016	1	1	1	1	1	0	1	1	1	8
Park, H. C 2015	1	1	1	1	0	0	1	0	1	6
Sciacqua, A 2015	0	1	1	1	1	1	1	0	0	6
Chrispin, J 2014	1	1	1	1	1	1	1	0	0	7
Chuang, S. Y 2014	1	1	1	1	1	1	1	0	0	7
Mora, S 2014	0	1	1	1	1	1	1	1	1	8
Agarwal, S. K 2013	1	1	1	1	0	0	1	1	0	6
Karppi, J 2013	1	1	1	1	0	0	1	1	1	7
Patton, K. K 2009	1	1	1	1	0	0	1	1	1	7
Ravn, L. S 2008	1	1	1	1	0	0	1	1	0	6
